# Low-molecular-weight heparin in the prevention of venous thromboembolism among patients with acute intracerebral hemorrhage: A meta-analysis

**DOI:** 10.1371/journal.pone.0311858

**Published:** 2024-10-16

**Authors:** Haizheng Li, Zhiguo Wu, Hongyu Zhang, Baohua Qiu, Yajun Wang

**Affiliations:** 1 Department of intervention, Tianjin Medical University Baodi Hospital, Tianjin, China; 2 Department of Cardiovascular Medicine, Tianjin Medical University Baodi Hospital, Tianjin, China; Global Health Neurology Lab / NSW Brain Clot Bank, NSW Health Pathology / Liverpool Hospital and South West Sydney Local Health District / Neurovascular Imaging Lab, Clinical Sciences Stream, Ingham Institute, AUSTRALIA

## Abstract

**Objective:**

It remains unclear whether low-molecular-weight heparin (LMWH) is effective and safe for intracerebral hemorrhage (ICH) patients. This study presents a meta-analysis for elucidating effect of LMWH on preventing venous thromboembolism (VTE) among ICH patients.

**Methods:**

Articles were located by systematically searching PubMed, Embase, Web of Science, Cochrane Library, China National Knowledge Infrastructure (CNKI), WANFANG DATA, VIP, and SinoMed databases. The literature was independently screened by two authors, who also extracted data and conducted a qualitative evaluation. With regard to outcomes, their risk ratios (RRs) and 95% confidence intervals (CIs) were computed, and the findings were combined using the random effects model by using Mantel-Haenszel approach.

**Results:**

30 studies involving 2904 patients were analyzed and compared to control group. According to our findings, early low-dose LMWH, prophylaxis for VTE, was related to the markedly reduced deep vein thrombosis (DVT) (3.6% vs. 17.5%; RR, 0.25; 95% CI, 0.18–0.35; p-value<0.00001) and pulmonary embolism (PE) (0.4% vs. 3.2%; RR, 0.29; 95% CI, 0.14–0.57; p-value = 0.003), while the non-significantly increased hematoma progression (3.8% vs. 3.4%; RR, 1.06; 95% CI, 0.68–1.68; p-value = 0.79) and gastrointestinal bleeding (3.6% vs. 6.1%; RR, 0.63; 95% CI, 0.31–1.28; p-value = 0.20). Also, mortality (14.1% vs. 15.8%; RR, 0.90; 95% CI, 0.63–1.28; p-value = 0.55) did not show any significant difference in LMWH compared with control groups.

**Conclusions:**

Our meta-analysis suggested that early low-dose of LMWH are safe and effective in ICH patients. More extensive, multicenter, high-quality randomized clinical trials (RCTs) should be conducted to validate the findings and inform clinical practice.

## 1. Introduction

Among patients with intracerebral hemorrhage (ICH), venous thromboembolism (VTE) presents life-threatening consequences and represents a significant global health burden. One study reported that the risks of deep vein thrombosis (DVT) and pulmonary embolism (PE) in ICH patients are 2.4% and 1.1%, respectively [1]. In line with the American Heart Association (AHA) and American Stroke Association (ASA) guidelines, intermittent pneumatic compression (IPC) should be initiated upon admission (class I; level of evidence A), while unfractionated heparin (UFH) or low-molecular-weight heparin (LMWH) can be administered within 1–4 day after admission (class II; level of evidence B) [2]. However, the 2020 guidelines of the Heart and Stroke Foundation of Canada (HSFC) recommend starting LMWH after two days of admission (level of evidence B) [3]. Most of the guidelines that report the use of LMWH and UFH for preventing VTE among ICH patients offer weak recommendations with low-quality evidence [2–5]. However, the American Society of Hematology (ASH) 2018 guidelines provide a strong recommendation for ICH patients, based on moderate certainty of evidence [6]. In view of limited related evidence, only 16.5% of ICH patients receive prophylactic anticoagulation [7]. This is primarily because it is believed that these patients have a high risk of bleeding. Therefore, this work conducted an improved meta-analysis on recent studies (randomized or non-randomized) to elucidate the role of LMWH for VTE prevention in ICH patients. Additionally, the effect of LMWH on DVT, PE, hematoma progression, gastrointestinal bleeding, and mortality was systematically analyzed.

## 2. Methods

### 2.1 Search strategy and screening criteria

The judicious protocol describing specific objectives, search strategy, screening criteria, study quality evaluation, clinical outcomes, and statistical analysis was developed. The protocol was written according to reporting guidelines of Preferred Reporting Items for Systematic Reviews and Meta-analyses (PRISMA). Our protocol was registered in PROSPERO database (registration number: CRD42024525822). PubMed, Embase, Web of Science, Cochrane Library, China National Knowledge Infrastructure (CNKI), WANFANG DATA, VIP, and SinoMed databases were comprehensively searched from inception to November 2023, in addition to a systematic manual search of journal articles. S1 Table shows more details about queries. Further, studies below were included: (1) ICH patients; (2) intervention: LMWH treatment only or LMWH with mechanical treatment [Graduated Compression Stockings (GCS), Intermittent pneumatic compression (IPC)]; (3) comparison: mechanical treatment (GCS, IPC) or not; (4) primary outcomes: DVT, PE, hematoma progression; and secondary outcomes: gastrointestinal bleeding, and mortality; (5) study design: randomized clinical trials (RCTs) and cohort study; (6) publications whose full-texts could be obtained to screen and extract data. In order to remove any irrelevant and non-specific studies, studies below were excluded: (1) Studies involving patients with ICH caused by surgery, traumatic brain injury, or those with intracranial hemorrhages other than ICH (e.g., subarachnoid hemorrhage, traumatic intracerebral hemorrhage, subdural hematoma, or epidural hematoma); (2) UFH or combined antiplatelet drugs; (3) non-original studies (like review, case report, meta-analysis, or systematic review). Endnote X9 was used to exclude duplicates and screen the literature. Two authors independently selected and assessed the eligibility of English studies or those published in non-English language (namely, Chinese), and any discrepancy was resolved by a third author.

### 2.2 Data collection

Data pertaining to the study design, type of intracerebral hemorrhage, treatment option, dosage, time of onset of treatment, duration of treatment, methods of diagnosis of DVT/PE/hematoma progression, follow-up period, event number, and study participant number were obtained by two investigators. Any disagreement in the data extraction was resolved by adjudicating with the third investigator.

### 2.3 Quality evaluation

Qualitative evaluation of RCTs and non-randomized trials (non-RCTs) was conducted by two investigators using the revised Cochrane risk-of-bias tool (RoB 2) and the Newcastle-Ottawa Scale (high quality 7≤to≤9, moderate quality 4≤to≤6, and low quality <4), respectively. Any conflict in the quality assessment was interceded by a third author.

### 2.4 Endpoints

The primary study endpoints were asymptomatic and symptomatic DVT (diagnosed by clinical symptoms, Doppler ultrasound, venography, and magnetic resonance imaging), PE (diagnosed by clinical symptoms and computed tomography pulmonary angiography), and hematoma progression (diagnosed by clinical symptoms and computed tomography, defined as a ≥33% increase in hematoma volume). The secondary study endpoints were gastrointestinal bleeding and mortality.

### 2.5 Statistical analysis

Review Manager V.5.3 software (Cochrane Collaboration, London, UK) and stata (StataCorp. 2017. Stata Statistical Software: Release 14; StataCorp LLC, College Station, Texas, USA) were employed for data analysis. Continuous variables were described as means (standard deviations), while categorical variables were described as numbers (percentages). We used Mantel-Haenszel approach-based random-effects model for combining results. Effect size was determined by calculating risk ratio (RR) and 95% confidence intervals (CI). To evaluate heterogeneities among the studies, the Mantel-Haenszel method was employed, with p-value = 0.10 indicating statistical significance. Furthermore, Higgins’ I^2^ statistic was employed to compare the extent of heterogeneity (low heterogeneity ≤30%, moderate heterogeneity 30%< to ≤50%, high heterogeneity >50%). For primary endpoints, at least ten studies were conducted for DVT, PE, and hematoma progression. Small-study effects were detected by assessing Egger’s test and funnel plot. The trim-and-fill approach was applied to provide an estimate of the treatment effect adjusted for selection bias. Additionally, subgroup analyses based on study design, ICH type, and ICH treatment type (operation or not) were performed. Lastly, the differences among the subgroups were examined in a random-effects model.

## 3. Results

### 3.1 Study screening

**Fig 1:**
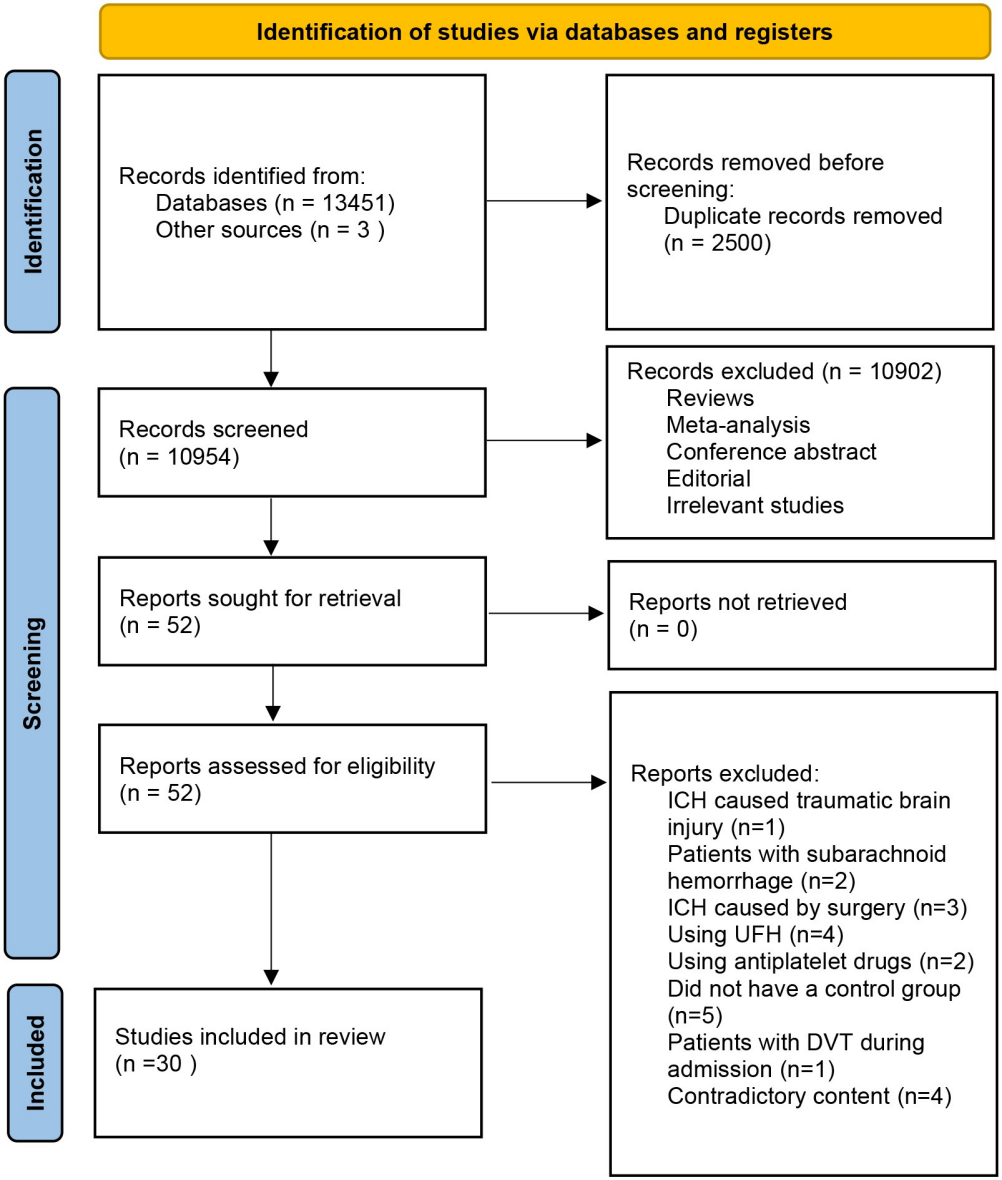
Flowchart showing the study inclusion and exclusion procedure.

Fig 1 exhibits our study screening procedure. There were altogether 13451 records obtained from databases search of citations; in addition, three more articles were found in additional sources. When duplicates were carefully eliminated, 10954 records were acquired and subsequently evaluated. After that, these articles were examined by title- and abstract-reading, with only 52 articles being retained for further study. The full-text of these selected 52 articles was assessed, among which, 30 were finally enrolled for the meta-analysis, while the remaining 22 were excluded. Table 1 summarizes the designs of these 30 qualified articles. The remaining 22 articles were excluded because of the following reasons: patients with traumatic ICH (one study) [8]; patients with subarachnoid hemorrhage (two studies) [9, 10]; patients with ICH due to surgery (three studies) [11–13]; use of UFH (four studies) [14–17]; use of antiplatelet drugs (two studies) [18, 19]; lack of control group (five studies) [20–24]; patients with DVT during admission (one study) [25]; contradictory content (four studies) [26–29].

**Table 1:** Features of enrolled articles.

Study	Design	Country	Type of ICH	Participants	Operation	Treatment	Control	Dosage	Time of onset of treatment	Duration of treatment	DVT diagnosis	PE diagnosis	Hematoma progression diagnosis	Follow-up duration
Zhao 2020	RCT	China	Unspecified ICH	70	Yes	LMWH	IPC+GCS	4000IU QD	Post operation 3rd day	≤10 days	Clinical	NR	NR	14 days
Ni 2018	Cohort study	China	Spontaneous ICH	104	No	LMWH (enoxaparin)+IPC	IPC	0.4ml QD	Admission 4th day	7 days	NE	NE	CT	7 days
Yu 2015	RCT	China	Unspecified ICH	103	No	LMWH	No LMWH	4000IU QD	NR	14 days	Doppler	CT	NR	14 days
Yin 2019	RCT	China	Unspecified ICH	86	No	LMWH+IPC+GCS	IPC+GCS	4000IU QD	Admission 3rd day	5 days	Doppler	NR	CT	14 days
Tang 2015	RCT	China	Hypertensive ICH	40	No	LMWH (nadroparin)	No LMWH	5000IU QD	Post-ICH 3rd/4th day	15 days	Doppler	CTA	CT	15 days
Mo 2021	RCT	China	Unspecified ICH	80	No	LMWH+IPC	IPC	57IU/kg QD	NR	14 days	NR	NR	NR	14 days
Xu 2019	RCT	China	Unspecified ICH	30	No	LMWH	No LMWH	0.4ml	NR	NR	Clinical	NE	NE	NR
Qian 2012	RCT	China	Hypertensive ICH	60	No	LMWH (nadroparin)+GCS	No LMWH	0.4ml QD	NR	14 days	Doppler	NR	CT	14 days
Feng 2021	RCT	China	Unspecified ICH	150	Yes	LMWH (nadroparin)+IPC	IPC	4100IU QD	Post operation 3rd day	10 days	Doppler	NR	NR	10 days
Li 2011	RCT	China	Hypertensive ICH	60	Yes	LMWH (nadroparin)+GCS	GCS	1025IU/10kg Q12H	Post operation 3rd day	10 days	Doppler	NE	CT	10 days
Jiang 2014	RCT	China	Hypertensive ICH	82	Yes	LMWH+IPC	IPC	4100IU QD	Post operation 3rd day	14 days	Doppler/MRI/Venography	NR	NR	21 days
Liu 2008	RCT	China	Hypertensive ICH	60	Yes	LMWH (nadroparin)+GCS	No LMWH	3500IU QD	NR	14 days	Doppler	NR	NR	14 days
Xia 2018	RCT	China	Hypertensive ICH	102	Yes	LMWH	No LMWH	9500/4750IU QD	NR	NR	Clinical	NR	NR	NR
Yang 2018	RCT	China	Hypertensive ICH	100	Yes	LMWH+IPC	No LMWH	0.2ml QD	Post operation 3rd day	≤14 days	Doppler	NE	NE	14 days
Wang 2015	RCT	China	Hypertensive ICH	106	Yes	LMWH+IPC+GCS	IPC+GCS	4100IU QD	Post operation 3rd day	Until TEG normal	Doppler	NE	NE	3 months
Yang 2010	RCT	China	Hypertensive ICH	99	Yes	LMWH	No LMWH	NR	Post operation 4th day	14 days	Doppler	NR	CT	14 days
Qin 2018	RCT	China	Hypertensive ICH	98	Yes	LMWH (nadroparin)+IPC	IPC	57IU/kg QD	Post operation 3rd day	14 days	Doppler/MRI/Venography	NR	CT	21 days
Guan 2019	RCT	China	Hypertensive ICH	92	Yes	LMWH (nadroparin)+IPC	IPC	0.4ml QD	Post operation 3rd day	14 days	Doppler	CTA	CT	6 months
Zhang 2017	Cohort study	China	Unspecified ICH	150	No	LMWH+IPC+GCS	IPC+GCS	4000IU QD	Admission 3rd day	NR	Doppler	NE	CT	14 days
Chen 2019	Cohort study	China	Unspecified ICH	120	No	LMWH	No LMWH	0.4ml	Admission 3rd day	14 days	NR	NR	NR	14 days
Gu 2014	Cohort study	China	Spontaneous ICH	94	No	LMWH (nadroparin)	IPC	0.4ml QD	Admission 4th day	10 days	Doppler	NE	CT	14 days
Wu 2022	Cohort study	China	Spontaneous ICH	91	Yes	LMWH (enoxaparin)+IPC	IPC	4000IU QD	Post operation 4th day	7–14 days	Doppler	NR	CT	28 days
Sun 2017	Cohort study	China	Hypertensive ICH	100	Yes	LMWH+IPC	IPC	4100IU QD	Post operation 3rd day	4 days	Clinical	NE	NE	4 days
Lu 2021	Cohort study	China	Unspecified ICH	82	Yes	LMWH+IPC	IPC	4100IU QD	Post operation 3rd day	14 days	NR	NE	NE	21 days
Yu 2022	RCT	China	Unspecified ICH	42	Yes	LMWH	No LMWH	3000IU QD	Admission 4th day	7d days	NR	NE	NE	7 days
Sui 2022	RCT	China	Unspecified ICH	50	Yes	LMWH+IPC	IPC	4100IU QD	Post operation 0 day	14d days	Clinical	NE	NE	14 days
Paciaroni 2020	RCT	Italy	Spontaneous ICH	73	No	LMWH (enoxaparin)	No LMWH	0.4 ml	NR	(10±1) days	Doppler	CTA	CT	90 days
Song 2021	Cohort study	China	Hypertensive ICH	98	No	LMWH+IPC+GCS	IPC+GCS	4000IU QD	NR	5 days	NR	NR	NR	5 days
Tetri 2008	Cohort study	Finland	Spontaneous ICH	407	No	LMWH (enoxaparin)	No LMWH	20mg QD	Post-ICH 2nd day	8 (5–12) days	NR	NR	CT	3 months
Orken 2009	RCT	Türkiye	Spontaneous ICH	75	No	LMWH (enoxaparin)	GCS	40mg QD	Admission 3rd day	NR	Doppler	CTA	CT	21 days

### 3.2 Study quality

**Fig 2:**
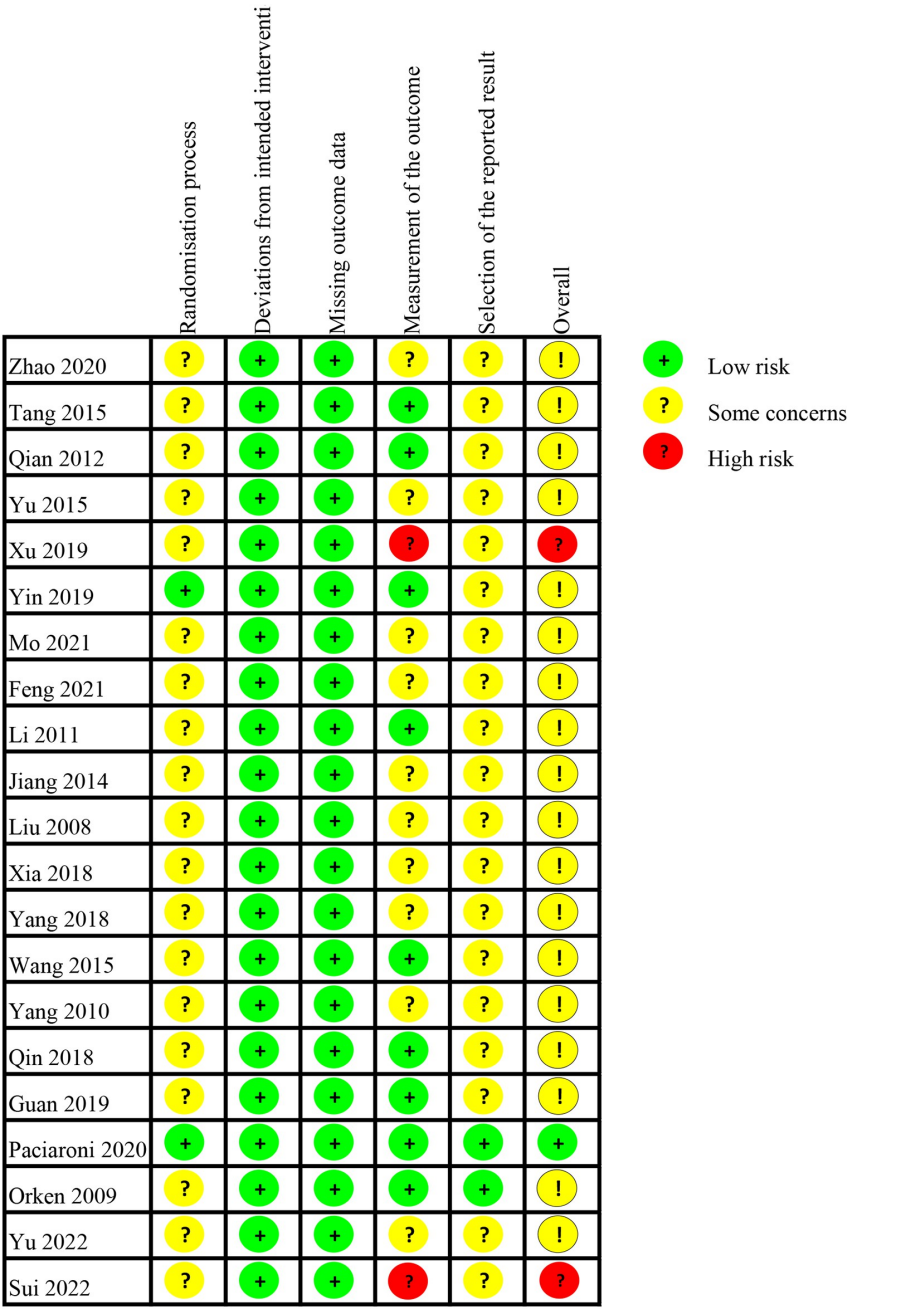
Quality assessment of randomized controlled trials.

Fig 2 and Table 2 outline quality data for those 30 included articles. Among the 21 RCTs, two were rated as high risk of overall bias since they used clinical diagnosis to measure the outcome. Also, most articles showed certain concerns in overall bias. Among the nine non-RCTs, eight were adjudged to be high quality, only one was of moderate quality.

**Table 2:** Quality evaluation of non-randomized controlled trials.

	Selection				Comparability	Outcome			
Study	Representativeness of the exposed cohort	Selection of the non exposed cohort	Ascertainment of exposure	Demonstration that outcome of interest was not present at start of study	Comparability of cohorts on the basis of the design or analysis	Assessment of outcome	Was follow-up long enough for outcomes to occur	Adequacy of follow up of cohorts	Total scores
Chen 2019	★	★	★	★	★★	☆	★	☆	7
Zhang 2017	★	★	★	★	★★	★	★	☆	8
Gu 2014	★	★	★	★	★★	★	★	☆	8
Wu 2022	★	★	★	★	★★	★	★	☆	8
Sun 2017	★	★	★	★	★★	☆	★	☆	7
Lu 2021	★	★	★	★	★★	☆	★	☆	7
Tetri 2008	★	★	★	★	☆☆	★	★	☆	6
Song 2021	★	★	★	★	★★	☆	★	☆	7
Ni 2018	★	★	★	★	★★	★	★	☆	8

### 3.3 Study outcomes

Altogether 30 articles (RCTs and non-RCTs) and 2904 patients were analyzed. Amongst these studies, 13 involved hypertensive ICH patients, 6 included spontaneous ICH cases, while 11 involved unspecified ICH patients. Also, 16 studies enrolled patients after ICH operation. Thromboprophylaxis was initiated on the 3rd to 4th day after hospitalization or ICH operation and continued for 14 days in most studies. In the majority of included studies, the dosing regimen of LMWH was generally 0.4 ml daily (enoxaparin 4000 IU per day, nadroparin 4100 IU per day).

**Fig 3:**
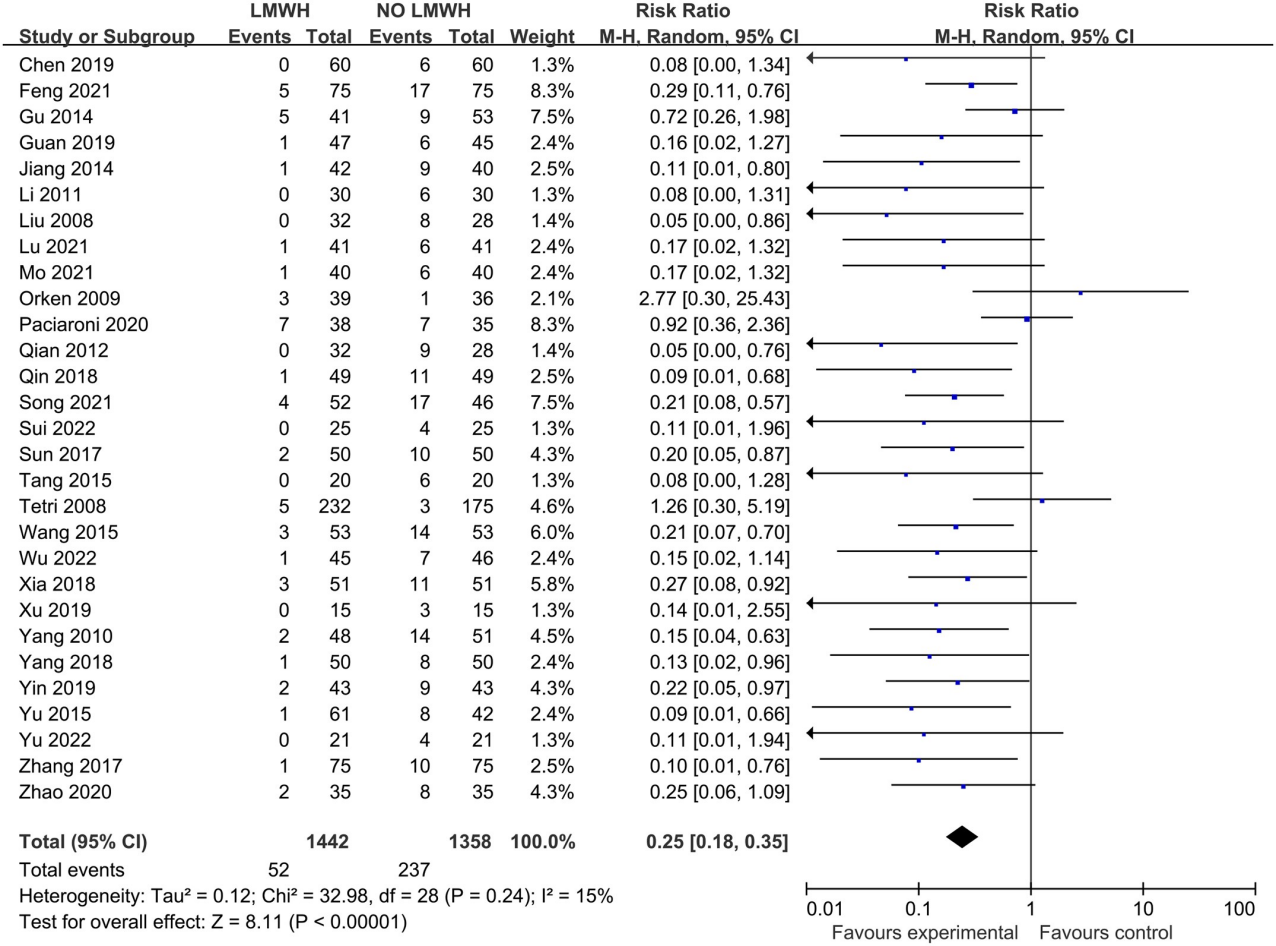
Effect of LMWH on DVT.

**Fig 4:**
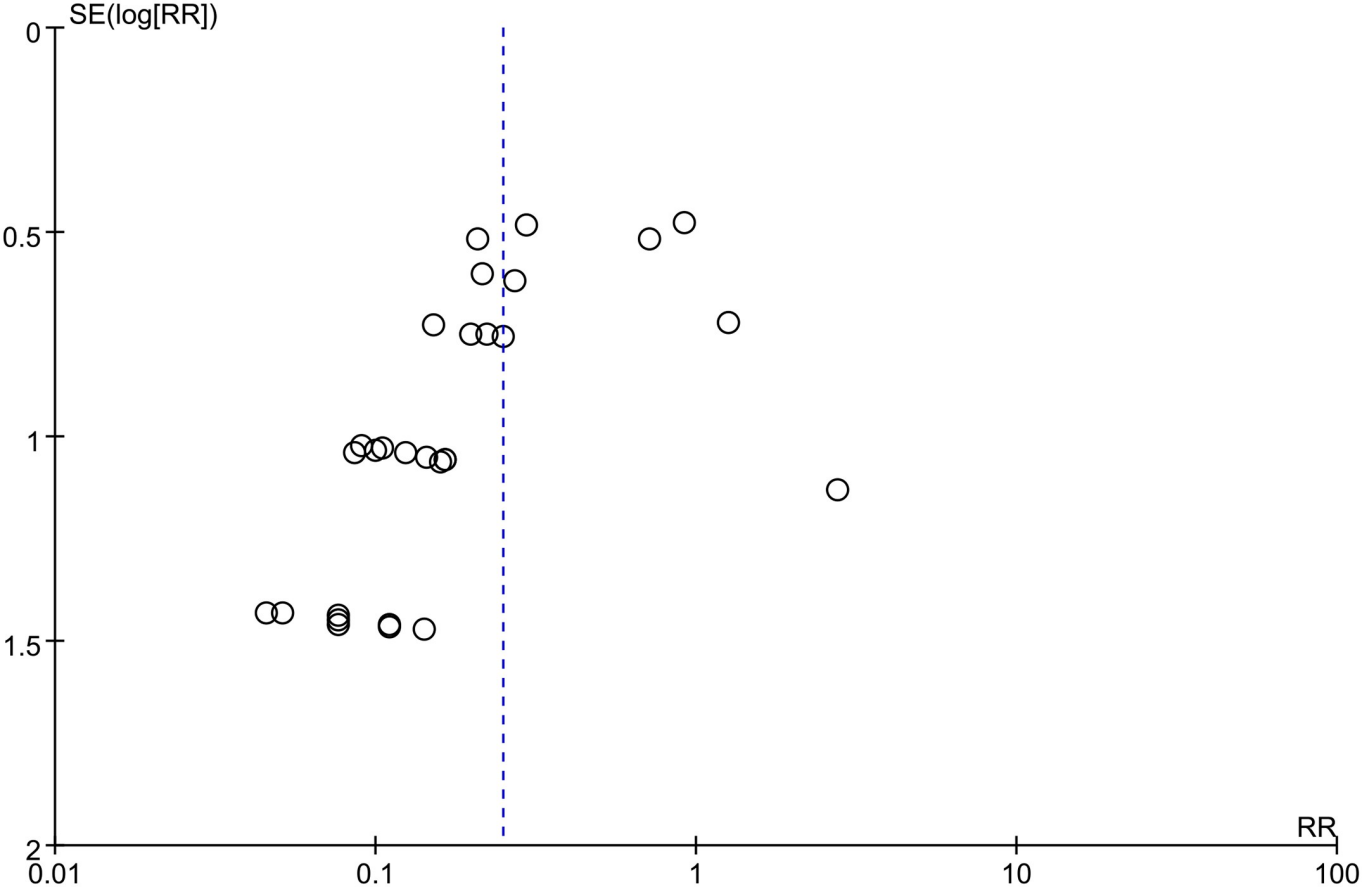
Funnel plot illustrating the role of LMWH in DVT.

There was no obvious heterogeneity for the effect of LMWH on DVT among 29 studies involving 2800 patients (I^2 =^ 15%; p-value = 0.24). In comparison with control group, LMWH group showed the significantly reduced DVT (3.7% vs. 17.5%; RR, 0.25; 95% CI, 0.18–0.35; p-value<0.00001) (Fig 3). Meanwhile, asymmetry could be seen from funnel plot of DVT (Egger’s test, p-value = 0.001) (Fig 4). The Filled meta-analysis performed by trim-and-fill method involved 29 articles, which conformed to initial analysis. S1–S3 Figs summaries the subgroup analysis results.

**Fig 5:**
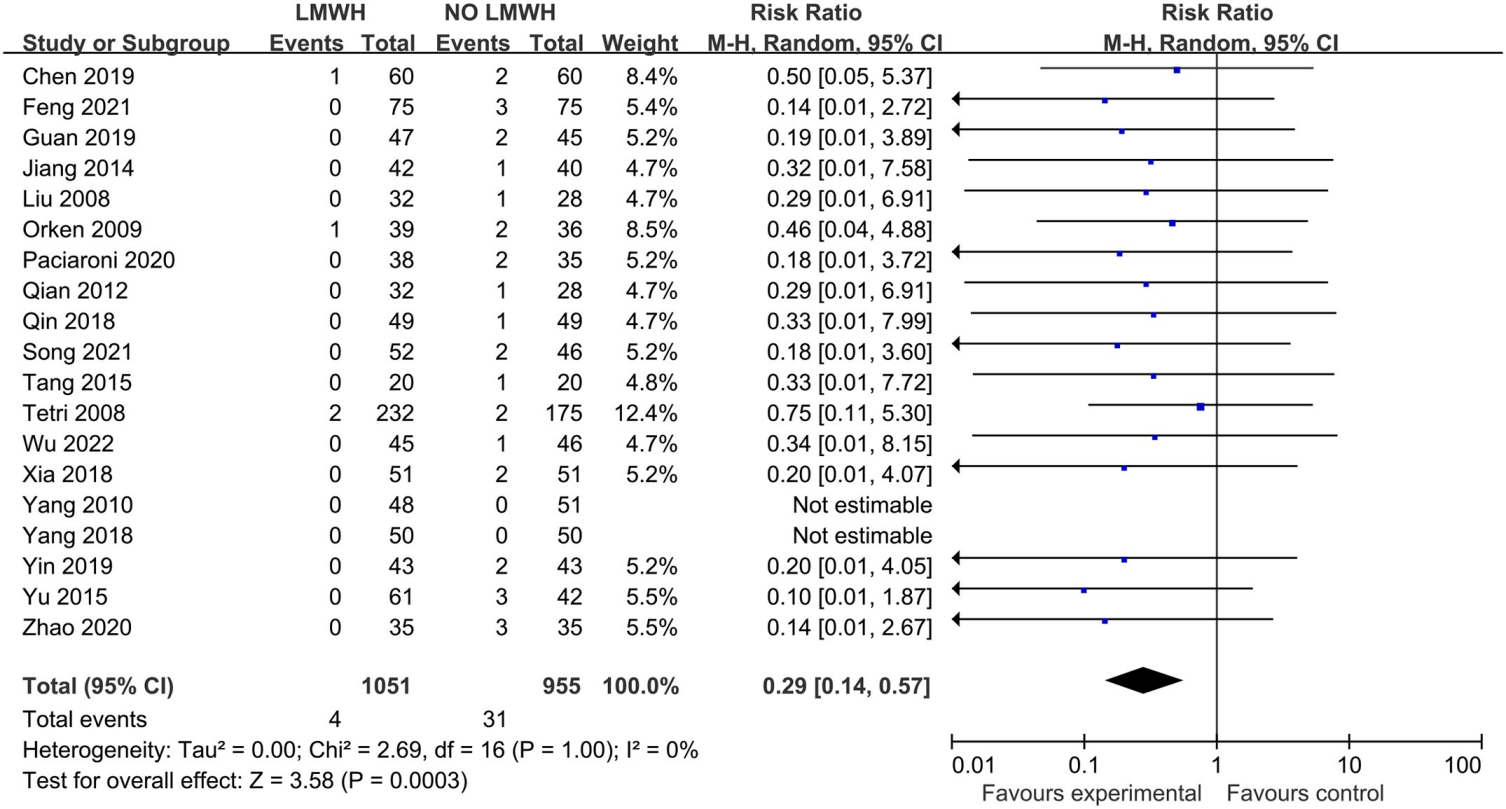
Effect of LMWH on PE.

**Fig 6:**
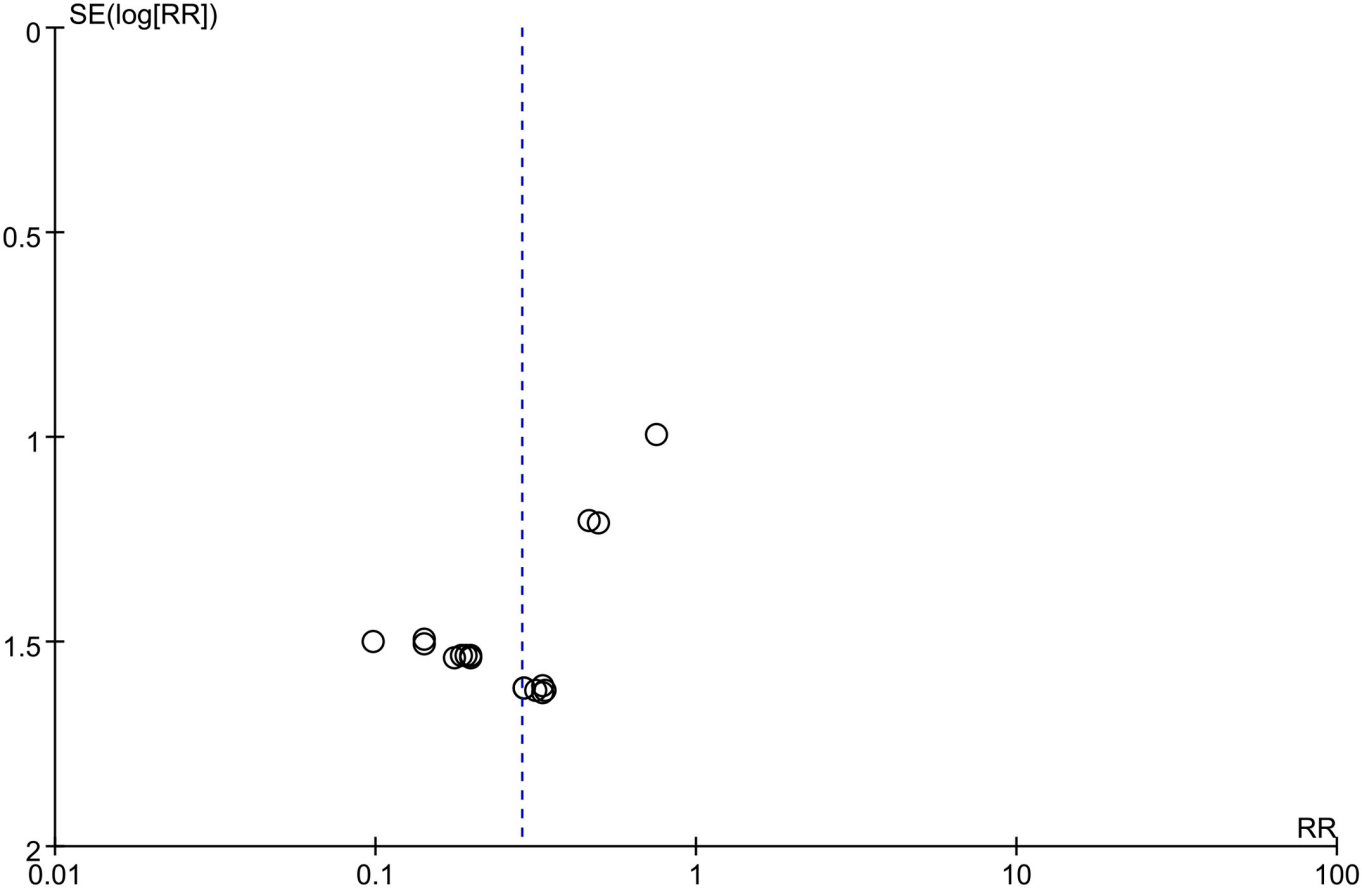
Funnel plot illustrating the role of LMWH in PE.

The effect of LMWH on PE, investigated based on 17 studies that involved 1807 patients, suggested the absence of obvious heterogeneity across those enrolled articles (I^2 =^ 0%; p-value = 1.00). LMWH group demonstrated significantly reduced PE in comparison with control group (0.4% vs. 3.2%; RR, 0.29; 95% CI, 0.14–0.57; p-value = 0.0003) (Fig 5). The funnel plot analysis of PE showed an asymmetrical shape (Egger’s test, p-value = 0.001) (Fig 6). The Filled meta-analysis performed by trim-and-fill method involved 17 articles, which conformed to initial analysis. S4–S6 Figs summarizes the subgroup analyses.

**Fig 7:**
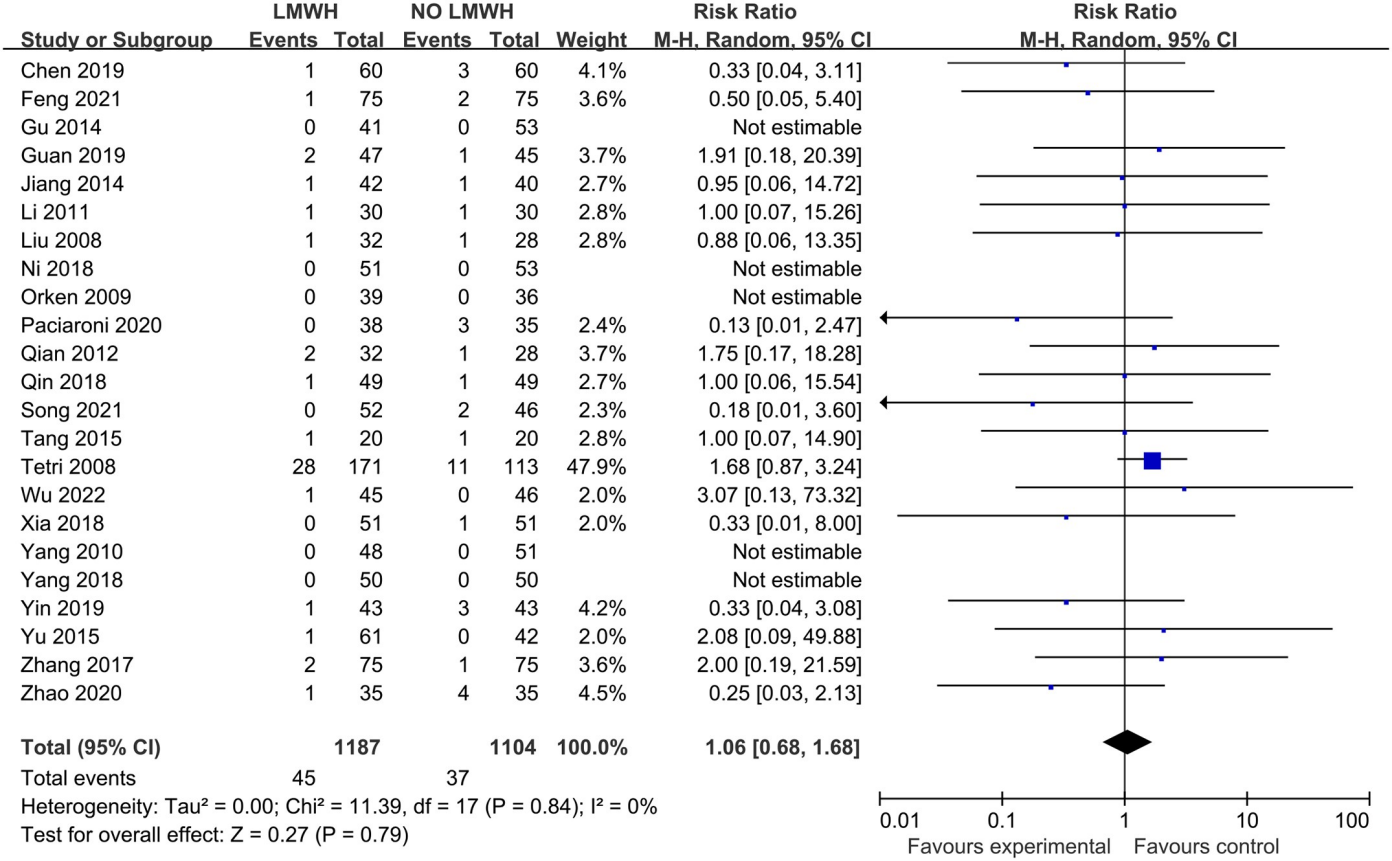
Effect of LMWH on hematoma progression.

**Fig 8:**
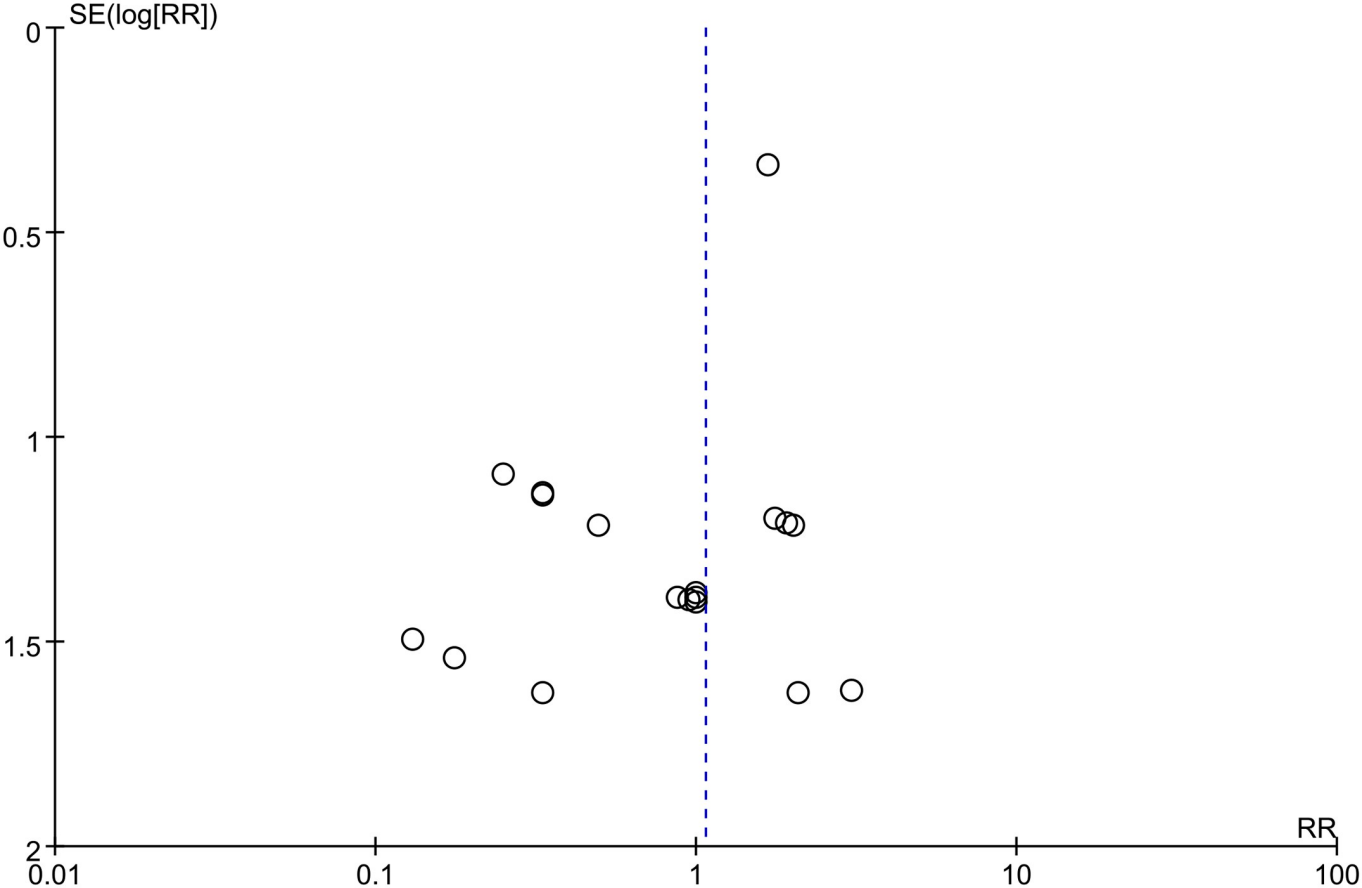
Funnel plot illustrating the role of LMWH in hematoma progression.

The effect of LMWH on hematoma progression was evaluated from 18 studies with 1819 patients and obvious heterogeneity was not found across diverse studies (I^2 =^ 0%; p-value = 0.84). Further, in comparison with control group, LMWH group exhibited the non-significant increase in hematoma progression (3.8% vs. 3.4%; RR, 1.06; 95% CI, 0.68–1.68; p-value = 0.79) (Fig 7). Asymmetry was observed from funnel plot of hematoma progression (Egger’s test, p-value = 0.03) (Fig 8). The Filled meta-analysis performed by trim-and-fill method involved 18 articles, which conformed to initial analysis. S7–S9 Figs displays the subgroup analyses.

**Fig 9:**
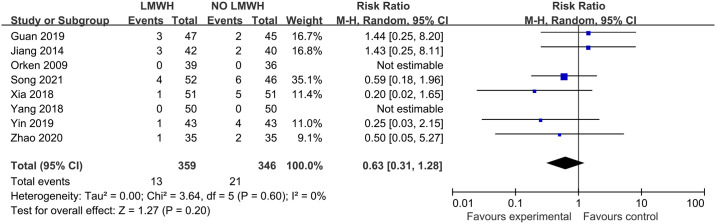
Effect of LMWH on gastrointestinal bleeding.

The meta-analysis of six studies with 530 patients on the effect of LMWH on gastrointestinal bleeding indicated the absence of obvious heterogeneity across our enrolled articles (I^2 =^ 0%; p-value = 0.60). The LMWH group showed a non-significant rise in gastrointestinal bleeding (3.6% vs. 6.1%; RR, 0.63; 95% CI, 0.31–1.28; p-value = 0.20) in comparison with control group (Fig 9). Subgroup analyses were summarized in S10–S12 Figs.

**Fig 10:**
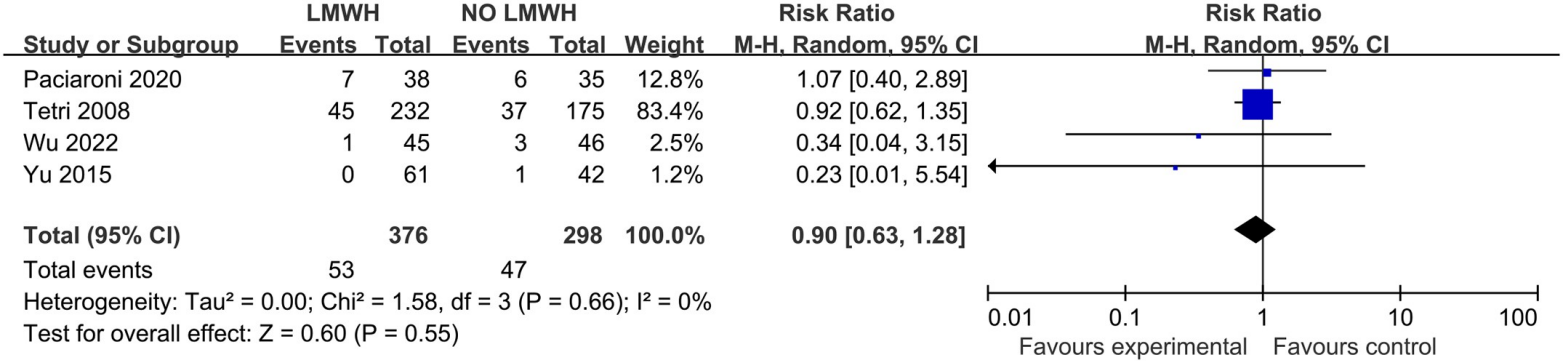
Effect of LMWH on mortality.

This work summarized the effect of LMWH on mortality in four articles involving 674 patients, and obvious heterogeneity was not observed across these articles (I^2 =^ 0%; p-value = 0.66). Further, mortality did not show any significant difference in LMWH versus control groups (14.1% vs. 15.8%; RR, 0.90; 95% CI, 0.63–1.28; p-value = 0.55) (Fig 10). The subgroup analyses were summarized in S13–S15 Figs.

## 4. Discussion

The clinical management of ICH patients, who develop DVT, is inconsistent due to the use of anticoagulant agents that may cause hematoma progression among the patients. Therefore, effective prophylaxis of VTE is needed in ICH patients. Generally, most of the guidelines provide weak recommendations with low quality evidence about using low-dose LMWH for ICH patients to prevent VTE [2–5]. Our findings demonstrate that in ICH patients, LMWH prophylaxis for VTE is related to the markedly reduced DVT and PE and the non-significantly increased hematoma progression, and gastrointestinal bleeding. One of the limitations of anticoagulant agents in ICH patients is the increased risk of mortality. However, our data showed that mortality did not exhibit any significant difference between the LMWH and control groups.

According to the results of our meta-analysis, LMWH was initiated on the 3rd to 4th day after admission or operation and continued for 14 days in most studies. The dosing regimen of LMWH was generally 0.4 ml daily (enoxaparin 4000 IU per day, nadroparin 4100 IU per day). Consequently, early administration of low-dose LMWH is both effective and safe in ICH patients. This information may assist doctors in making clinical decisions. To further support these findings, we analyzed three subgroups, which strengthened our findings. Because we believed that whether ICH patients accept surgical treatment may affect the outcome, so we performed a subgroup analysis. In a previous meta-analysis on 4 articles, applying heparin for preventing VTE is related to the markedly reduced PE [30]. The present data is in good agreement with a recent meta-analysis that includes 28 studies and 3697 patients and demonstrates that heparin is effective and safe among ICH patients [31]. A study with 68 ICH patients suggests that heparin initiation on day two is correlated with the more markedly reduced PE than on day four or day ten [14]. However, more investigations are warranted for determining the best way to prevent VTE among ICH patients.

Although our meta-analysis offers valuable information on LMWH in preventing venous thromboembolism among ICH patients, there are certain limitations, e.g., small sample size, inclusion of non-randomized studies, concerns about overall bias, relatively short follow-up duration, and lack of long-term outcomes. Hence, additional large, multicenter, high-quality RCTs are necessary to validate the findings and inform clinical practice.

## Supporting information

S1 Checklist PRISMA 2020 checklist. (DOCX)

S1 File ALL studies identified in the literature search. (XLSX)

S1 Table Search details. (DOCX)

S2 Table List of articles included in the meta-analysis. (DOCX)

S3 Table Summary of outcomes in enrolled study. (DOCX)

S4 Table Quality assessment of randomized controlled trials. (DOCX)

S1 Fig Subgroup analysis stratified by study design: Effect of LMWH on DVT. (TIF)

S2 Fig Subgroup analysis stratified by ICH type: Effect of LMWH on DVT. (TIF)

S3 Fig Subgroup analysis stratified by ICH treatment type: Effect of LMWH on DVT. (TIF)

S4 Fig Subgroup analysis stratified by study design: Effect of LMWH on PE. (TIF)

S5 Fig Subgroup analysis stratified by ICH type: Effect of LMWH on PE. (TIF)

S6 Fig Subgroup analysis stratified by ICH treatment type: Effect of LMWH on PE. (TIF)

S7 Fig Subgroup analysis stratified by study design: Role of LMWH in hematoma progression. (TIF)

S8 Fig Subgroup analysis stratified by ICH type: Effect of LMWH on hematoma progression. (TIF)

S9 Fig Subgroup analysis stratified by ICH treatment type: Effect of LMWH on hematoma progression. (TIF)

S10 Fig Subgroup analysis stratified by study design: Effect of LMWH on gastrointestinal bleeding. (TIF)

S11 Fig Subgroup analysis stratified by ICH type: Effect of LMWH on gastrointestinal bleeding. (TIF)

S12 Fig Subgroup analysis stratified by ICH treatment type: Effect of LMWH on gastrointestinal bleeding. (TIF)

S13 Fig Subgroup analysis based on study design: Effect of LMWH on mortality. (TIF)

S14 Fig Subgroup analysis based on ICH type: Effect of LMWH on mortality. (TIF)

S15 Fig Subgroup analysis based on ICH treatment type: Effect of LMWH on mortality. (TIF)
