# Supplementary material for: Low-molecular-weight heparin in the prevention of venous thromboembolism among patients with acute intracerebral hemorrhage: A meta-analysis
Source: PLoS One. 2024 Oct 16;19(10):e0311858. doi: 10.1371/journal.pone.0311858 (PMC11482721; doi:10.1371/journal.pone.0311858)
Supplement: S1 Table — (DOCX) [file pone.0311858.s003.docx]

**S1 Table. Search Details**

| #7 | #4 AND #5 AND #6 |
| --- | --- |
| #6 | #1 OR #2 OR #3 |
| #5 | (((((((((((((((((((((((((((Anticoagulant[MeSH Terms]) OR (anticoagulation[Title/Abstract])) OR (Anticoagulant therapy[Title/Abstract])) OR (Rivaroxaban[Title/Abstract])) OR (Edoxaban[Title/Abstract])) OR (Apixaban[Title/Abstract])) OR (Dabigatran[Title/Abstract])) OR (Heparin[Title/Abstract])) OR (Heparinoids[Title/Abstract])) OR (low-molecular-weight heparin[Title/Abstract])) OR (LMWH[Title/Abstract])) OR (Unfractioned heparin[Title/Abstract])) OR (UFH[Title/Abstract])) OR (Warfarin[Title/Abstract])) OR (Anticoagulant Drug[Title/Abstract])) OR (Drug, Anticoagulant[Title/Abstract])) OR (Anticoagulant Agents[Title/Abstract])) OR (Agents, Anticoagulant[Title/Abstract])) OR (Anticoagulation Agents[Title/Abstract])) OR (Agents, Anticoagulation[Title/Abstract])) OR (Anticoagulant Drugs[Title/Abstract])) OR (Drugs, Anticoagulant[Title/Abstract])) OR (Anticoagulant Agent[Title/Abstract])) OR (Agent, Anticoagulant[Title/Abstract])) OR (Anticoagulant[Title/Abstract])) OR (Indirect Thrombin Inhibitors[Title/Abstract])) OR (Inhibitors, Indirect Thrombin[Title/Abstract])) OR (Thrombin Inhibitors, Indirect[Title/Abstract]) |
| #4 | ((((((((((((((((((((((((((deep venous thrombosis[MeSH Terms]) OR (DVT[Title/Abstract])) OR (Phlebothrombosis[Title/Abstract])) OR (Phlebothromboses[Title/Abstract])) OR (Thrombosis, Venous[Title/Abstract])) OR (Thromboses, Venous[Title/Abstract])) OR (Venous Thromboses[Title/Abstract])) OR (Deep Vein Thrombosis[Title/Abstract])) OR (Deep Vein Thromboses[Title/Abstract])) OR (Thromboses, Deep Vein[Title/Abstract])) OR (Vein Thromboses, Deep[Title/Abstract])) OR (Vein Thrombosis, Deep[Title/Abstract])) OR (Deep-Venous Thrombosis[Title/Abstract])) OR (Deep-Venous Thromboses[Title/Abstract])) OR (Thromboses, Deep-Venous[Title/Abstract])) OR (Thrombosis, Deep-Venous[Title/Abstract])) OR (Deep-Vein Thrombosis[Title/Abstract])) OR (Deep-Vein Thromboses[Title/Abstract])) OR (Thromboses, Deep-Vein[Title/Abstract])) OR (Thrombosis, Deep-Vein[Title/Abstract])) OR (Thrombosis, Deep Vein[Title/Abstract])) OR (Deep Venous Thrombosis[Title/Abstract])) OR (Deep Venous Thromboses[Title/Abstract])) OR (Thromboses, Deep Venous[Title/Abstract])) OR (Thrombosis, Deep Venous[Title/Abstract])) OR (Venous Thromboses, Deep[Title/Abstract])) OR (Venous Thrombosis, Deep[Title/Abstract]) |
| #3 | ((((((((((((((((((((((((((((Stroke[MeSH Terms]) OR (Strokes[Title/Abstract])) OR (Cerebrovascular Accident[Title/Abstract])) OR (Cerebrovascular Accidents[Title/Abstract])) OR (CVA (Cerebrovascular Accident[Title/Abstract]))) OR (CVAs (Cerebrovascular Accident[Title/Abstract]))) OR (Cerebrovascular Apoplexy[Title/Abstract])) OR (Apoplexy, Cerebrovascular[Title/Abstract])) OR (Vascular Accident, Brain[Title/Abstract])) OR (Brain Vascular Accident[Title/Abstract])) OR (Brain Vascular Accidents[Title/Abstract])) OR (Vascular Accidents, Brain[Title/Abstract])) OR (Cerebrovascular Stroke[Title/Abstract])) OR (Cerebrovascular Strokes[Title/Abstract])) OR (Stroke, Cerebrovascular[Title/Abstract])) OR (Strokes, Cerebrovascular[Title/Abstract])) OR (Apoplexy[Title/Abstract])) OR (Cerebral Stroke[Title/Abstract])) OR (Cerebral Strokes[Title/Abstract])) OR (Stroke, Cerebral[Title/Abstract])) OR (Strokes, Cerebral[Title/Abstract])) OR (Stroke, Acute[Title/Abstract])) OR (Acute Stroke[Title/Abstract])) OR (Acute Strokes[Title/Abstract])) OR (Strokes, Acute[Title/Abstract])) OR (Cerebrovascular Accident, Acute[Title/Abstract])) OR (Acute Cerebrovascular Accident[Title/Abstract])) OR (Acute Cerebrovascular Accidents[Title/Abstract])) OR (Cerebrovascular Accidents, Acute[Title/Abstract]) |
| #2 | ((((((((((((((Intracranial hemorrhage[MeSH Terms]) OR (Intracranial haemorrhage[Title/Abstract])) OR (Brain hemorrhage[Title/Abstract])) OR (Brain haemorrhage[Title/Abstract])) OR (Hemorrhages, Intracranial[Title/Abstract])) OR (Intracranial Hemorrhage[Title/Abstract])) OR (Hemorrhage, Intracranial[Title/Abstract])) OR (Posterior Fossa Hemorrhage[Title/Abstract])) OR (Hemorrhage, Posterior Fossa[Title/Abstract])) OR (Hemorrhages, Posterior Fossa[Title/Abstract])) OR (Posterior Fossa Hemorrhages[Title/Abstract])) OR (Brain Hemorrhage[Title/Abstract])) OR (Brain Hemorrhages[Title/Abstract])) OR (Hemorrhage, Brain[Title/Abstract])) OR (Hemorrhages, Brain[Title/Abstract]) |
| #1 | ((((((((((((((((((((((((((Cerebral hemorrhage[MeSH Terms]) OR (Cerebral haemorrhage[Title/Abstract])) OR (Intracerebral haemorrhage[Title/Abstract])) OR (ICH[Title/Abstract])) OR (Hemorrhage, Cerebrum[Title/Abstract])) OR (Cerebrum Hemorrhage[Title/Abstract])) OR (Cerebrum Hemorrhages[Title/Abstract])) OR (Hemorrhages, Cerebrum[Title/Abstract])) OR (Cerebral Parenchymal Hemorrhage[Title/Abstract])) OR (Cerebral Parenchymal Hemorrhages[Title/Abstract])) OR (Hemorrhage, Cerebral Parenchymal[Title/Abstract])) OR (Hemorrhages, Cerebral Parenchymal[Title/Abstract])) OR (Parenchymal Hemorrhage, Cerebral[Title/Abstract])) OR (Parenchymal Hemorrhages, Cerebral[Title/Abstract])) OR (Intracerebral Hemorrhage[Title/Abstract])) OR (Hemorrhage, Intracerebral[Title/Abstract])) OR (Hemorrhages, Intracerebral[Title/Abstract])) OR (Intracerebral Hemorrhages[Title/Abstract])) OR (Hemorrhage, Cerebral[Title/Abstract])) OR (Cerebral Hemorrhages[Title/Abstract])) OR (Hemorrhages, Cerebral[Title/Abstract])) OR (Brain Hemorrhage, Cerebral[Title/Abstract])) OR (Brain Hemorrhages, Cerebral[Title/Abstract])) OR (Cerebral Brain Hemorrhage[Title/Abstract])) OR (Cerebral Brain Hemorrhages[Title/Abstract])) OR (Hemorrhage, Cerebral Brain[Title/Abstract])) OR (Hemorrhages, Cerebral Brain[Title/Abstract]) |
