# Supplementary material for: Low-molecular-weight heparin in the prevention of venous thromboembolism among patients with acute intracerebral hemorrhage: A meta-analysis
Source: PLoS One. 2024 Oct 16;19(10):e0311858. doi: 10.1371/journal.pone.0311858 (PMC11482721; doi:10.1371/journal.pone.0311858)
Supplement: S2 Table — (DOCX) [file pone.0311858.s004.docx]

| **S2 Table. List of articles included in the meta-analysis** | |
| --- | --- |
| Number | Article |
| 1 | Paciaroni M, Agnelli G, Alberti A, Becattini C, Guercini F, Martini G, et al. PREvention of VENous Thromboembolism in Hemorrhagic Stroke Patients - PREVENTIHS Study: A Randomized Controlled Trial and a Systematic Review and Meta-Analysis. Eur Neurol. 2020;83(6):566-575. |
| 2 | Orken DN, Kenangil G, Ozkurt H, Guner C, Gundogdu L, Basak M, et al. Prevention of deep venous thrombosis and pulmonary embolism in patients with acute intracerebral hemorrhage. Neurologist. 2009 Nov;15(6):329-31. |
| 3 | Song H, Men H, Song C, Feng H, Tan H, Zuo S. Low-molecular-weight heparin reduces the formation of lower limb deep venous thrombosis in patients with hypertensive intracerebral hemorrhage. Am J Transl Res. 2021 Sep 15;13(9):10509-10516. |
| 4 | Tetri S, Hakala J, Juvela S, Saloheimo P, Pyhtinen J, Rusanen H, et al. Safety of low-dose subcutaneous enoxaparin for the prevention of venous thromboembolism after primary intracerebral haemorrhage. Thromb Res. 2008;123(2):206-12. |
| 5 | 赵俊霞,刘颖超.低分子肝素钙对脑出血下肢深静脉血栓形成的预防效果.深圳中西医结合杂志,2020,30(14):187-188. |
| 6 | 倪杰,钱健,王路娜,等.低分子肝素预防脑出血后深静脉血栓有效性和安全性研究.中国卒中杂志,2018,13(06):562-566. |
| 7 | 于红春,杨伟,苏莉.低剂量低分子肝素钠预防脑出血患者下肢深静脉血栓形成临床体会.脑与神经疾病杂志,2015,23(04):299-301. |
| 8 | 尹丽鹤,刘秋武,董延慧.分析低分子肝素钙对脑出血下肢深静脉血栓的预防作用.中国继续医学教育,2019,11(05):109-111. |
| 9 | 唐平,王少丹,梁彦涛,等.抗凝治疗预防高血压脑出血继发栓塞性疾病的疗效及安全性研究.现代医药卫生,2015,31(24):3719-3721. |
| 10 | 莫岸辉. 脑出血患者采用低分子肝素治疗对降低下肢深静脉血栓形成率的作用. 首都食品与医药,2021,28(10):60-61. |
| 11 | 许鹏,张军,黄妍,等.研究早期康复干预联合低分子肝素对脑出血患者深静脉血栓形成的预防作用.世界最新医学信息文摘,2019,19(A2):113-115. |
| 12 | 钱聪,王义荣.应用低分子肝素在高血压脑出血患者预防深静脉血栓形成的疗效.浙江临床医学,2012,14(9):1030-1032 |
| 13 | 陈敢.早期低分子肝素应用预防脑出血患者下肢深静脉血栓的疗效观察.双足与保健,2019,28(01):33-34. |
| 14 | 张焕.早期低分子肝素应用预防脑出血患者下肢深静脉血栓的疗效观察.现代实用医学,2017,29(12):1563-1564. |
| 15 | 顾双双,张均,韩玲,等. 早期那屈肝素预防脑出血患者深静脉血栓形成的安全性. 国际脑血管病杂志,2014,22(12):902-906. |
| 16 | 冯景,张晓丽.低分子肝素钙预防脑出血术后下肢深静脉血栓的效果及对凝血指标的影响.中外医学研究,2021,19(08):25-27. |
| 17 | 佘晓岚,王剑芳.低分子肝素钙预防脑出血术后下肢深静脉血栓的效果及对凝血指标的影响分析.中文科技期刊数据库（文摘版）医药卫生,2022(8):13-15 |
| 18 | 李爱静,刘海华.低分子肝素钙注射液预防高血压脑出血术后患者下肢深静脉血栓形成的疗效和安全性探讨.中风与神经疾病杂志,2011,28(03):253-254. |
| 19 | 管敏武,王新东,胡国平,等.低分子肝素预防高血压继发性脑室出血外引流术后静脉血栓栓塞症的临床研究.现代实用医学,2019,31(10):1356-1358. |
| 20 | 蒋涛,冯家龙,刘中洪,等.低分子肝素预防幕上高血压脑出血术后下肢深静脉血栓形成的效果观察.武警医学,2014,25(11):1095-1097. |
| 21 | 隋松涛,范凤,孙金波.低剂量低分子肝素钙在老年脑出血患者下肢深静脉血栓预防中的作用.中国老年学杂志,2022,42(22):5429-5431. |
| 22 | 刘伟国,刘春萍,王丽,等.高血压脑出血手术后深静脉血栓栓塞症预防性治疗.山西医科大学学报,2008,(08):738-740. |
| 23 | 夏传星.高血压脑出血术后使用低分子肝素预防下肢深静脉血栓形成的效果观察.双足与保健,2018,27(16):15-16.DOI:10.19589/j.cnki.issn1004-6569.2018.16.015. |
| 24 | 羊飞龙,许伍环.气压治疗联合低分子肝素预防高血压脑出血术后深静脉血栓的临床评价.世界最新医学信息文摘,2018,18(04):22-23+26. |
| 25 | 王涛.血栓弹力图指导下应用低分子肝素钙预防高血压脑出血术后DVT效果观察.山东医药,2015,55(32):46-47. |
| 26 | 杨明,夏志民.应用低分子肝素钙预防高血压脑出血术后深静脉血栓的临床研究.中国社区医师(医学专业),2010,12(26):32. |
| 27 | 秦征东,葛欣.应用低分子肝素钙预防高血压性脑出血术后深静脉血栓形成的疗效和安全性.中国医药,2018,13(04):541-544. |
| 28 | 吴华勇,蒋冰洁,戴伟民. 低分子肝素对自发性脑实质内出血术后下肢深静脉血栓形成的预防价值. 中华急诊医学杂志,2022,31(8):1131-1134. |
| 29 | 卢泽均,徐聪,罗嘉文. 低分子肝素钙预防脑出血手术患者下肢深静脉血栓形成的临床效果观察. 医药前沿,2021,11(33):77-78. |
| 30 | 孙卫国,朱琳. 高血压脑出血术后使用低分子肝素预防下肢深静脉血栓形成的效果. 双足与保健,2017,26(21):122-123. |
