# Supplementary material for: Low-molecular-weight heparin in the prevention of venous thromboembolism among patients with acute intracerebral hemorrhage: A meta-analysis
Source: PLoS One. 2024 Oct 16;19(10):e0311858. doi: 10.1371/journal.pone.0311858 (PMC11482721; doi:10.1371/journal.pone.0311858)
Supplement: S3 Table — (DOCX) [file pone.0311858.s005.docx]

| **S3 Table. Summary of outcomes in enrolled study** | | | | | | | | | | |
| --- | --- | --- | --- | --- | --- | --- | --- | --- | --- | --- |
| Study | DVT | | PE | | Hematoma progression | | Gastrointestinal bleeding | | Death | |
|  | LMWH group | Control group | LMWH group | Control group | LMWH group | Control group | LMWH group | Control group | LMWH group | Control group |
| Zhao 2020 | 2 | 8 | 0 | 3 | 1 | 4 | 0 | 2 | - | - |
| Ni 2018 | - | - | - | - | 0 | 0 | - | - | - | - |
| Yu 2015 | 1 | 8 | 0 | 3 | 1 | 0 | - | - | 0 | 1 |
| Yin 2019 | 2 | 9 | 0 | 2 | 1 | 3 | 0 | 4 | - | - |
| Tang 2015 | 0 | 6 | 0 | 1 | 1 | 1 | - | - | - | - |
| Mo 2021 | 1 | 6 | - | - | - | - | - | - | - | - |
| Xu 2019 | 0 | 3 | - | - | - | - | - | - | - | - |
| Qian 2012 | 0 | 9 | 0 | 1 | 2 | 1 | - | - | - | - |
| Feng 2021 | 5 | 17 | 0 | 3 | 1 | 2 | - | - | - | - |
| Li 2011 | 0 | 6 | - | - | 1 | 1 | - | - | - | - |
| Jiang 2014 | 1 | 9 | 0 | 1 | 1 | 1 | 0 | 2 | - | - |
| Liu 2008 | 0 | 8 | 0 | 1 | 1 | 1 | - | - | - | - |
| Xia 2018 | 3 | 11 | 0 | 2 | 0 | 1 | 0 | 5 | - | - |
| Yang 2018 | 1 | 8 | 0 | 0 | 0 | 0 | 0 | 0 | - | - |
| Wang 2015 | 3 | 14 | - | - | - | - | - | - | - | - |
| Yang 2010 | 2 | 14 | 0 | 0 | 0 | 0 | - | - | - | - |
| Qin 2018 | 1 | 11 | 0 | 1 | 1 | 1 | - | - | - | - |
| Guan 2019 | 1 | 6 | 0 | 2 | 2 | 1 | 0 | 2 | - | - |
| Zhang 2017 | 1 | 10 | - | - | 2 | 1 | - | - | - | - |
| Chen 2019 | 0 | 6 | 1 | 2 | 1 | 3 | - | - | - | - |
| Gu 2014 | 5 | 9 | - | - | 0 | 0 | - | - | - | - |
| Wu 2022 | 1 | 7 | 0 | 1 | 1 | 0 | - | - | 1 | 3 |
| Sun 2017 | 2 | 10 | - | - | - | - | - | - | - | - |
| Lu 2021 | 1 | 6 | - | - | - | - | - | - | - | - |
| Yu 2022 | 0 | 4 | - | - | - | - | - | - | - | - |
| Sui 2022 | 0 | 4 | - | - | - | - | - | - | - | - |
| Paciaroni 2020 | 7 | 7 | 0 | 2 | 0 | 3 | - | - | 7 | 6 |
| Song 2021 | 4 | 17 | 0 | 2 | 0 | 2 | 0 | 6 | - | - |
| Tetri 2008 | 5 | 3 | 2 | 2 | 28 | 11 | - | - | 45 | 37 |
| Orken 2009 | 3 | 1 | 1 | 2 | 0 | 0 | 1 | 0 | - | - |
| Data extractors: Haizheng Li and Yajun Wang. Date of data extraction: April 10, 2024. | | | | | | | | | | |
