# Supplementary material for: Low-molecular-weight heparin in the prevention of venous thromboembolism among patients with acute intracerebral hemorrhage: A meta-analysis
Source: PLoS One. 2024 Oct 16;19(10):e0311858. doi: 10.1371/journal.pone.0311858 (PMC11482721; doi:10.1371/journal.pone.0311858)
Supplement: S4 Table — (DOCX) [file pone.0311858.s006.docx]

| **S4 Table. Quality assessment of randomized controlled trials** | | | | | |  |
| --- | --- | --- | --- | --- | --- | --- |
| Study | Randomization process | Deviations from intended interventions | Mising outcome data | Measurement of the outcome | Selection of the reported result | Overall Bias |
| Zhao 2020 | Some concerns | Low | Low | Some concerns | Some concerns | Some concerns |
| Tang 2015 | Some concerns | Low | Low | Low | Some concerns | Some concerns |
| Qian 2012 | Some concerns | Low | Low | Low | Some concerns | Some concerns |
| Yu 2015 | Some concerns | Low | Low | Some concerns | Some concerns | Some concerns |
| Xu 2019 | Some concerns | Low | Low | High | Some concerns | High |
| Yin 2019 | Low | Low | Low | Low | Some concerns | Some concerns |
| Mo 2021 | Some concerns | Low | Low | Some concerns | Some concerns | Some concerns |
| Feng 2021 | Some concerns | Low | Low | Some concerns | Some concerns | Some concerns |
| Li 2011 | Some concerns | Low | Low | Low | Some concerns | Some concerns |
| Jiang 2014 | Some concerns | Low | Low | Some concerns | Some concerns | Some concerns |
| Liu 2008 | Some concerns | Low | Low | Some concerns | Some concerns | Some concerns |
| Xia 2018 | Some concerns | Low | Low | Some concerns | Some concerns | Some concerns |
| Yang 2018 | Some concerns | Low | Low | Some concerns | Some concerns | Some concerns |
| Wang 2015 | Some concerns | Low | Low | Low | Some concerns | Some concerns |
| Yang 2010 | Some concerns | Low | Low | Some concerns | Some concerns | Some concerns |
| Qin 2018 | Some concerns | Low | Low | Low | Some concerns | Some concerns |
| Guan 2019 | Some concerns | Low | Low | Low | Some concerns | Some concerns |
| Paciaroni 2020 | Low | Low | Low | Low | Low | Low |
| Orken 2009 | Some concerns | Low | Low | Low | Low | Some concerns |
| Yu 2022 | Some concerns | Low | Low | Some concerns | Some concerns | Some concerns |
| Sui 2022 | Some concerns | Low | Low | High | Some concerns | High |
