# Supplementary figures and images for: Low-molecular-weight heparin in the prevention of venous thromboembolism among patients with acute intracerebral hemorrhage: A meta-analysis
Source: PLoS One. 2024 Oct 16;19(10):e0311858. doi: 10.1371/journal.pone.0311858 (PMC11482721; doi:10.1371/journal.pone.0311858)

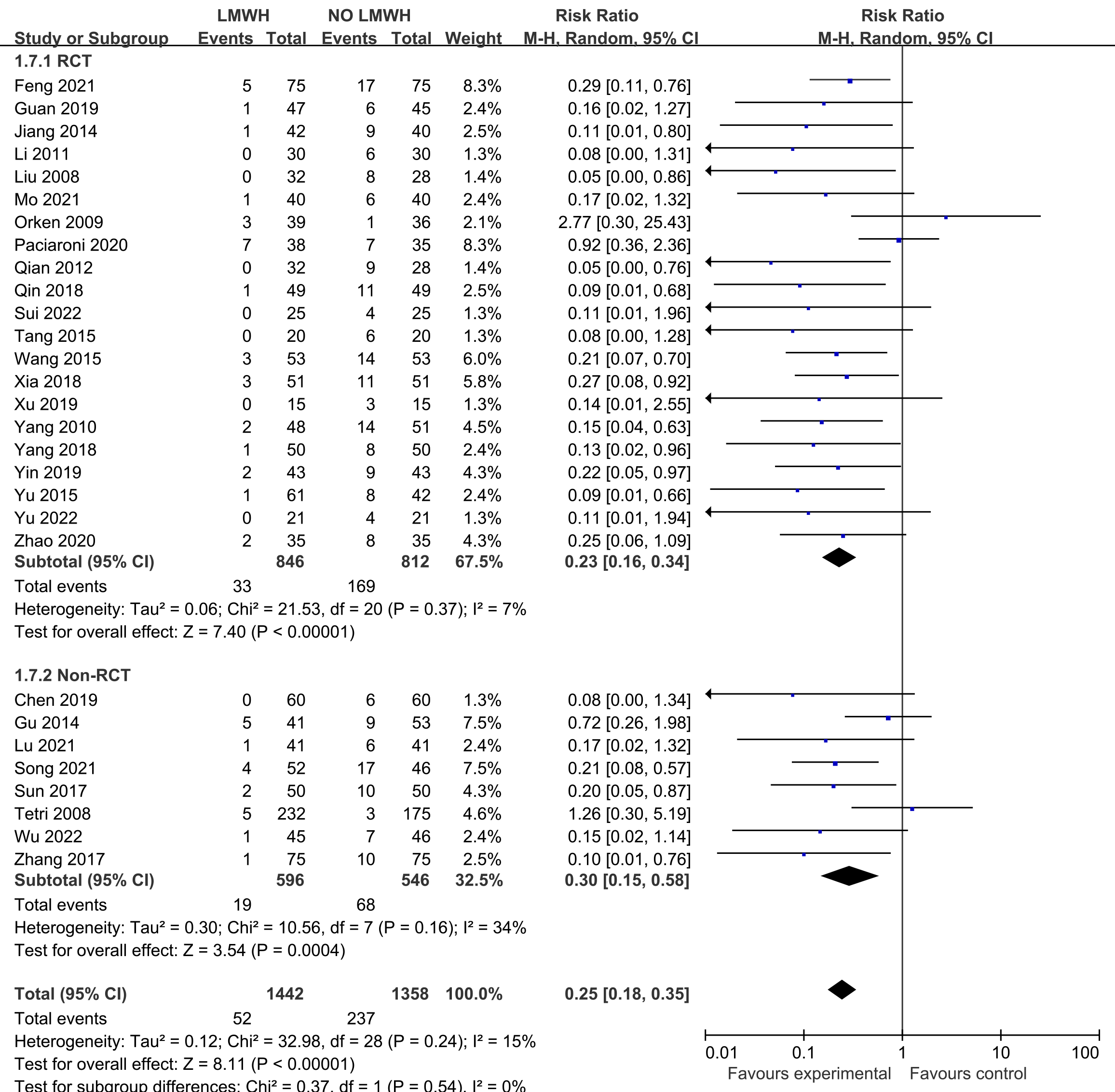

Supplement: S1 Fig — (TIF) [file pone.0311858.s007.tif]

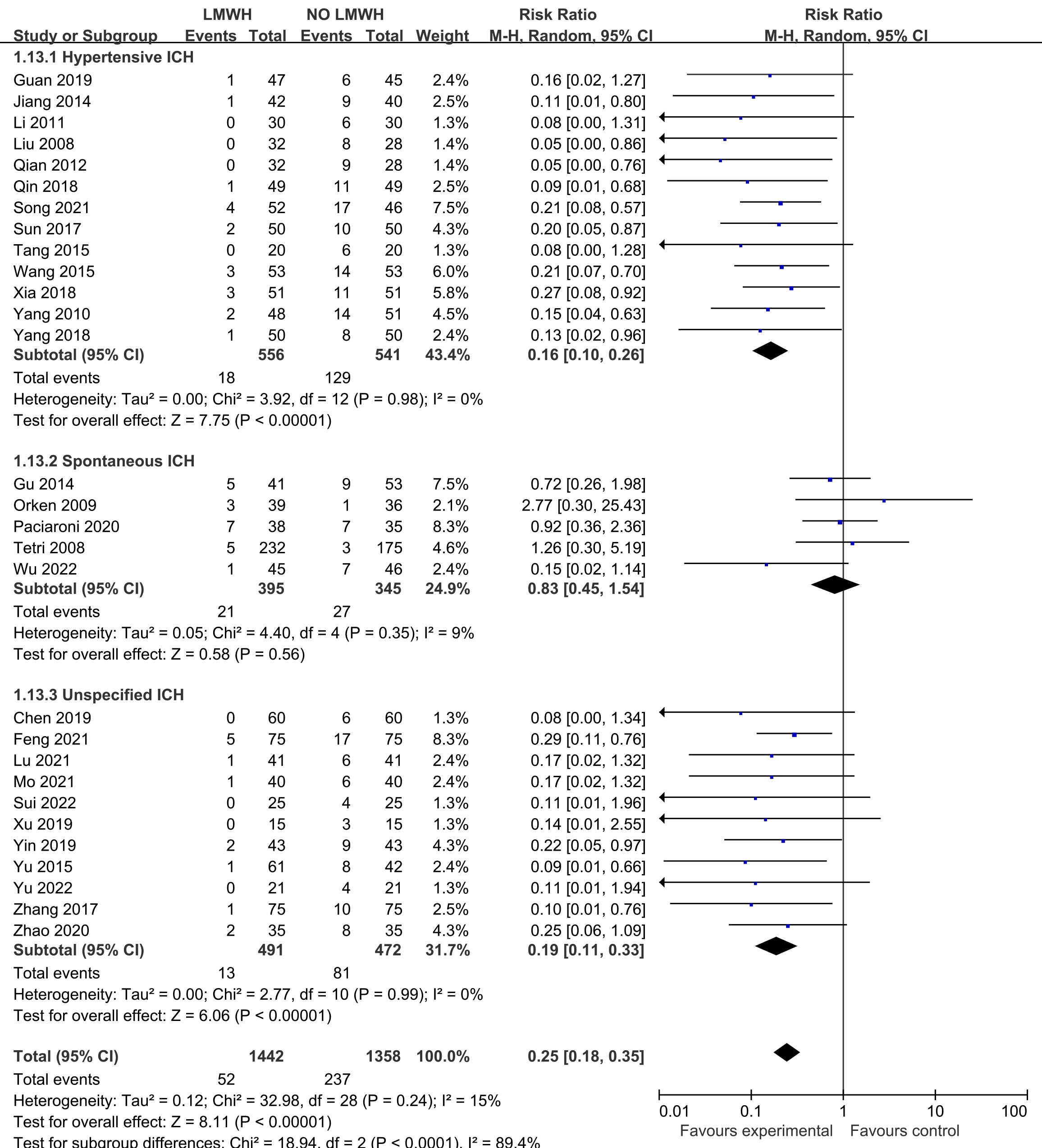

Supplement: S2 Fig — (TIF) [file pone.0311858.s008.tif]

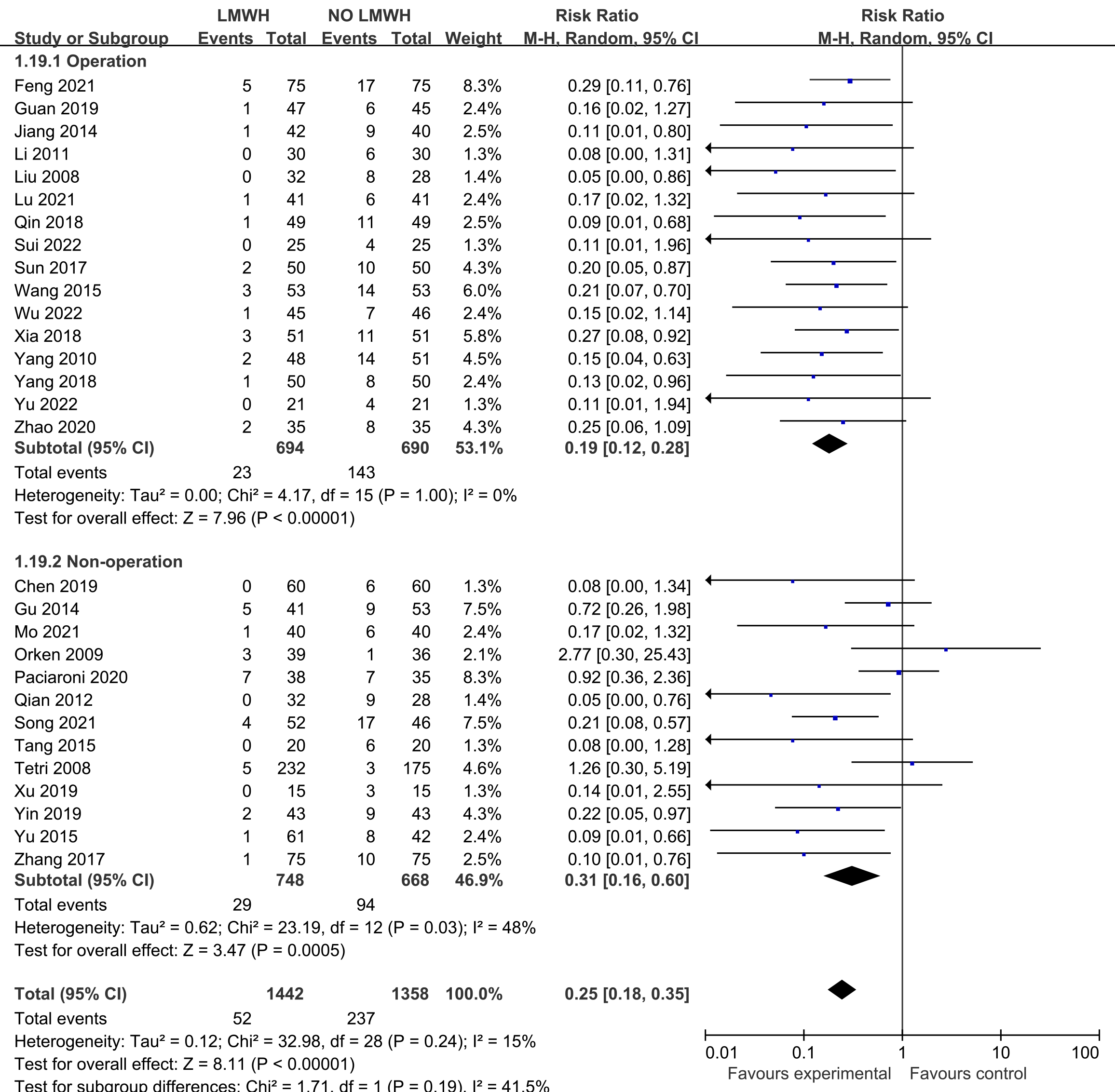

Supplement: S3 Fig — (TIF) [file pone.0311858.s009.tif]

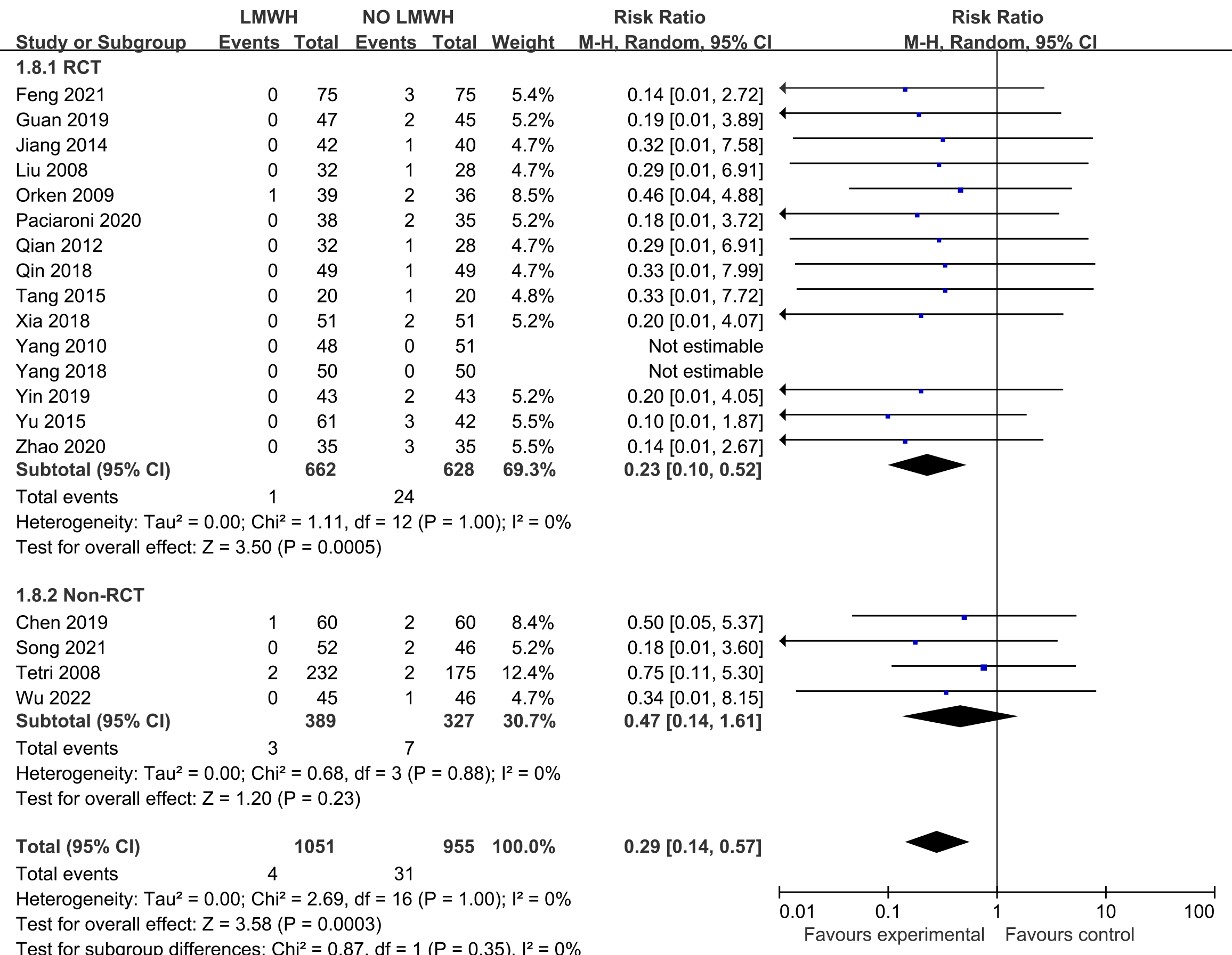

Supplement: S4 Fig — (TIF) [file pone.0311858.s010.tif]

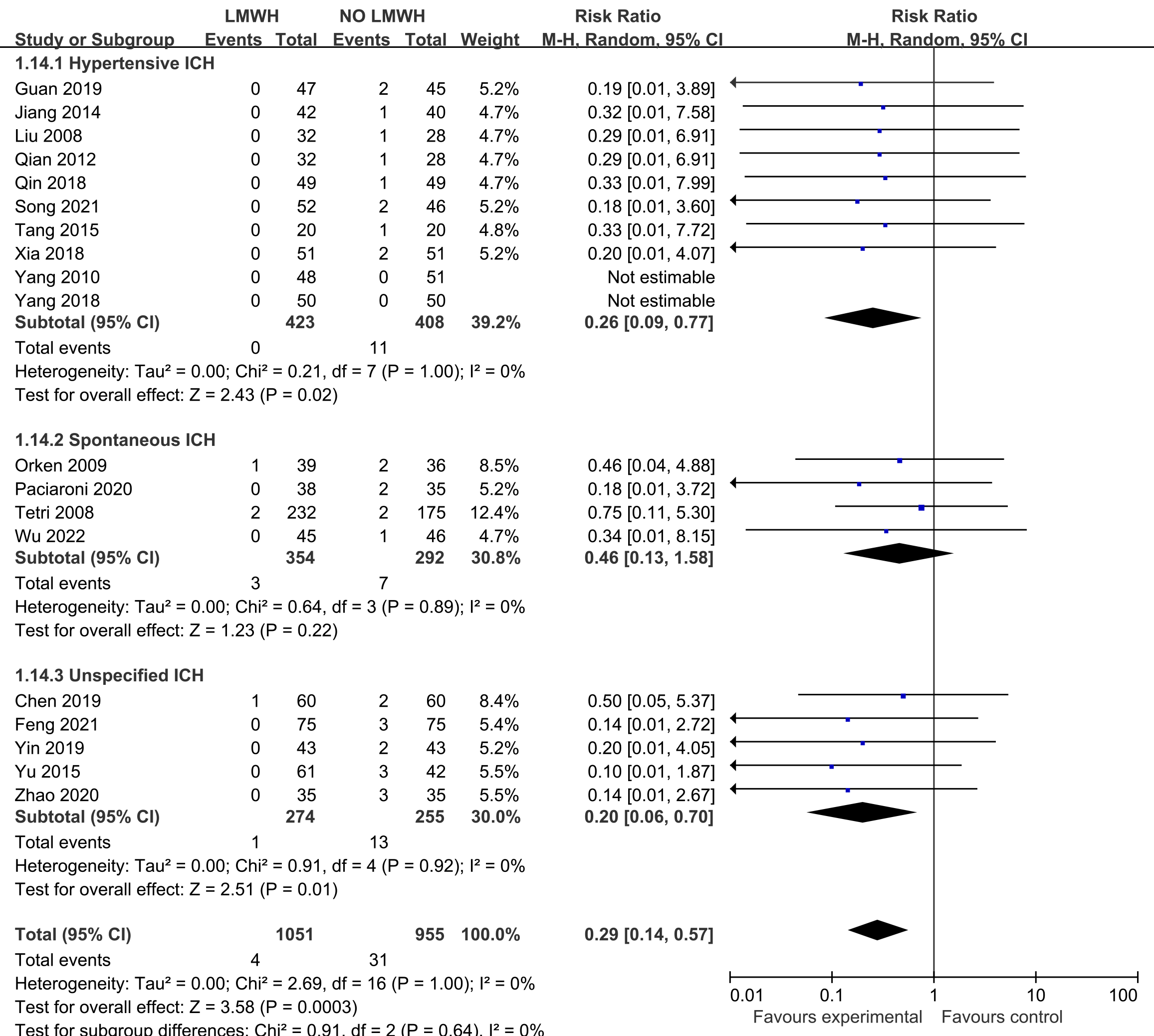

Supplement: S5 Fig — (TIF) [file pone.0311858.s011.tif]

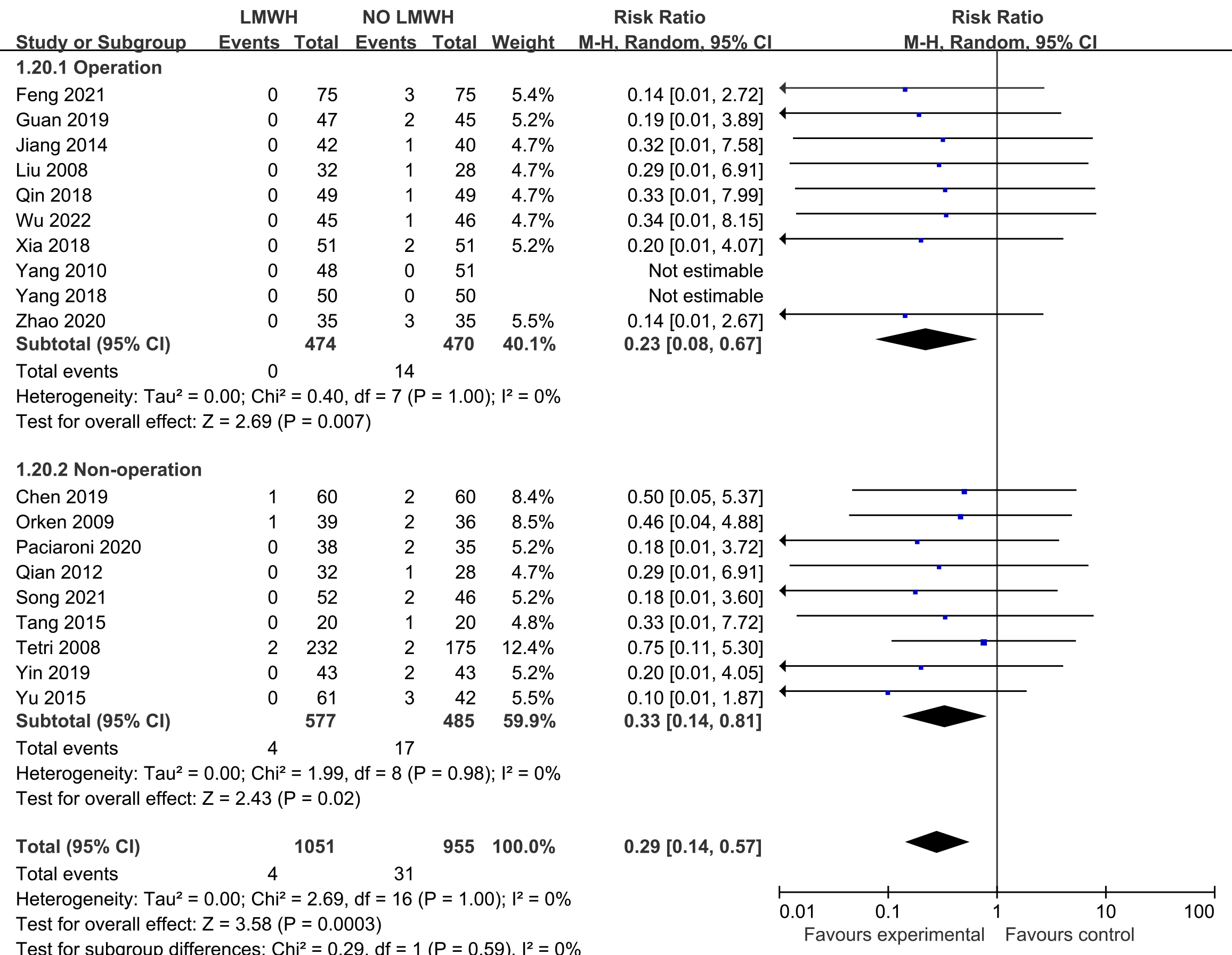

Supplement: S6 Fig — (TIF) [file pone.0311858.s012.tif]

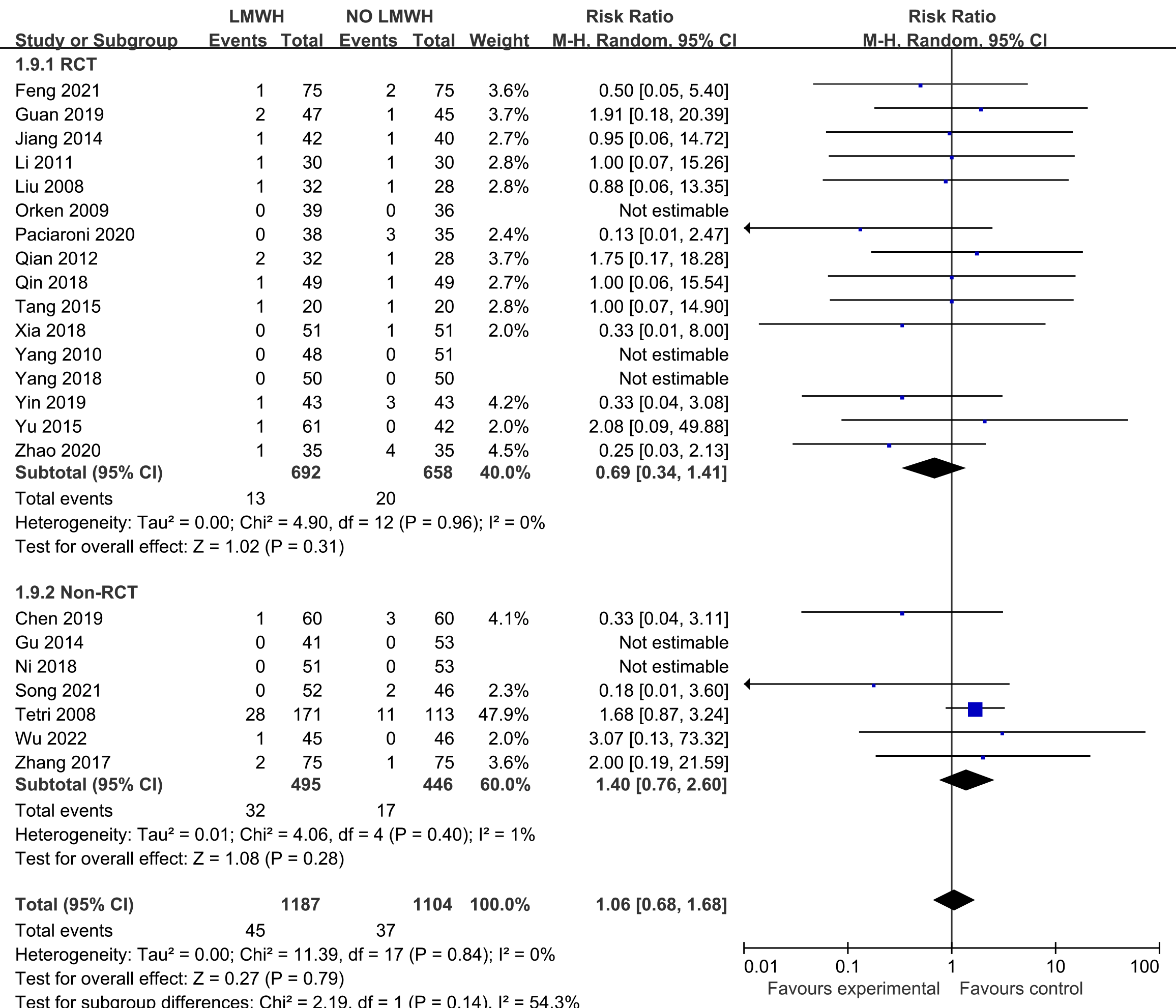

Supplement: S7 Fig — (TIF) [file pone.0311858.s013.tif]

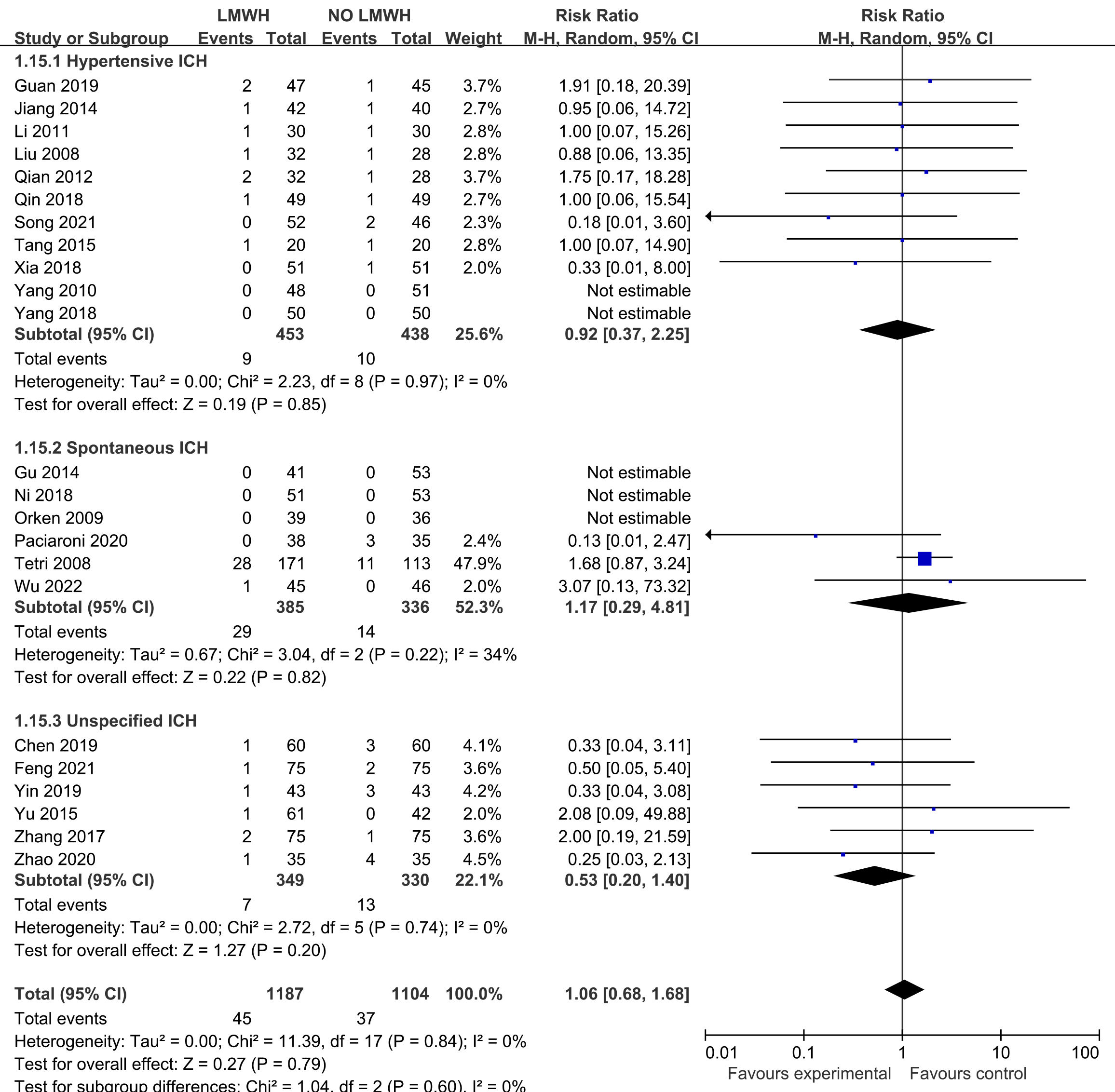

Supplement: S8 Fig — (TIF) [file pone.0311858.s014.tif]

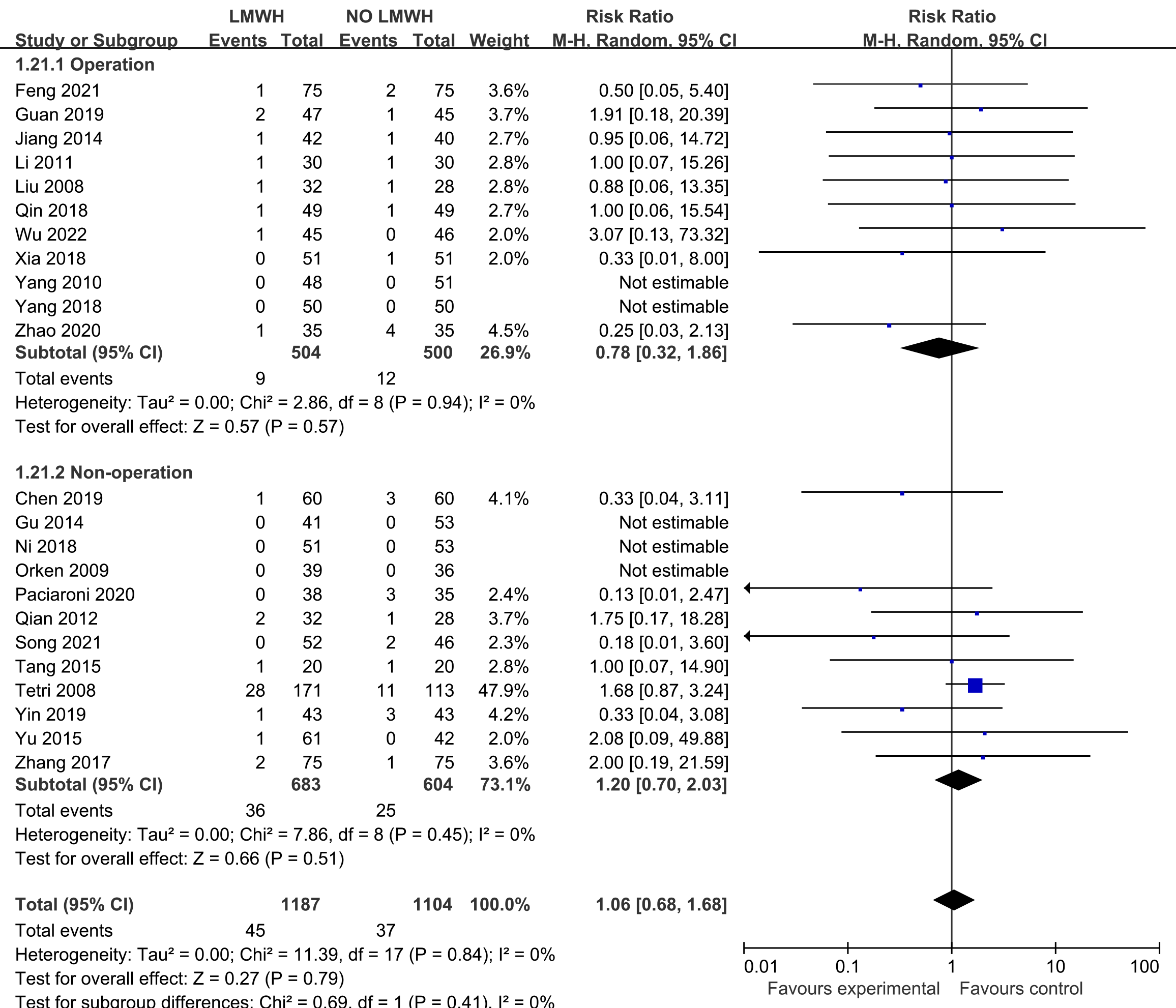

Supplement: S9 Fig — (TIF) [file pone.0311858.s015.tif]

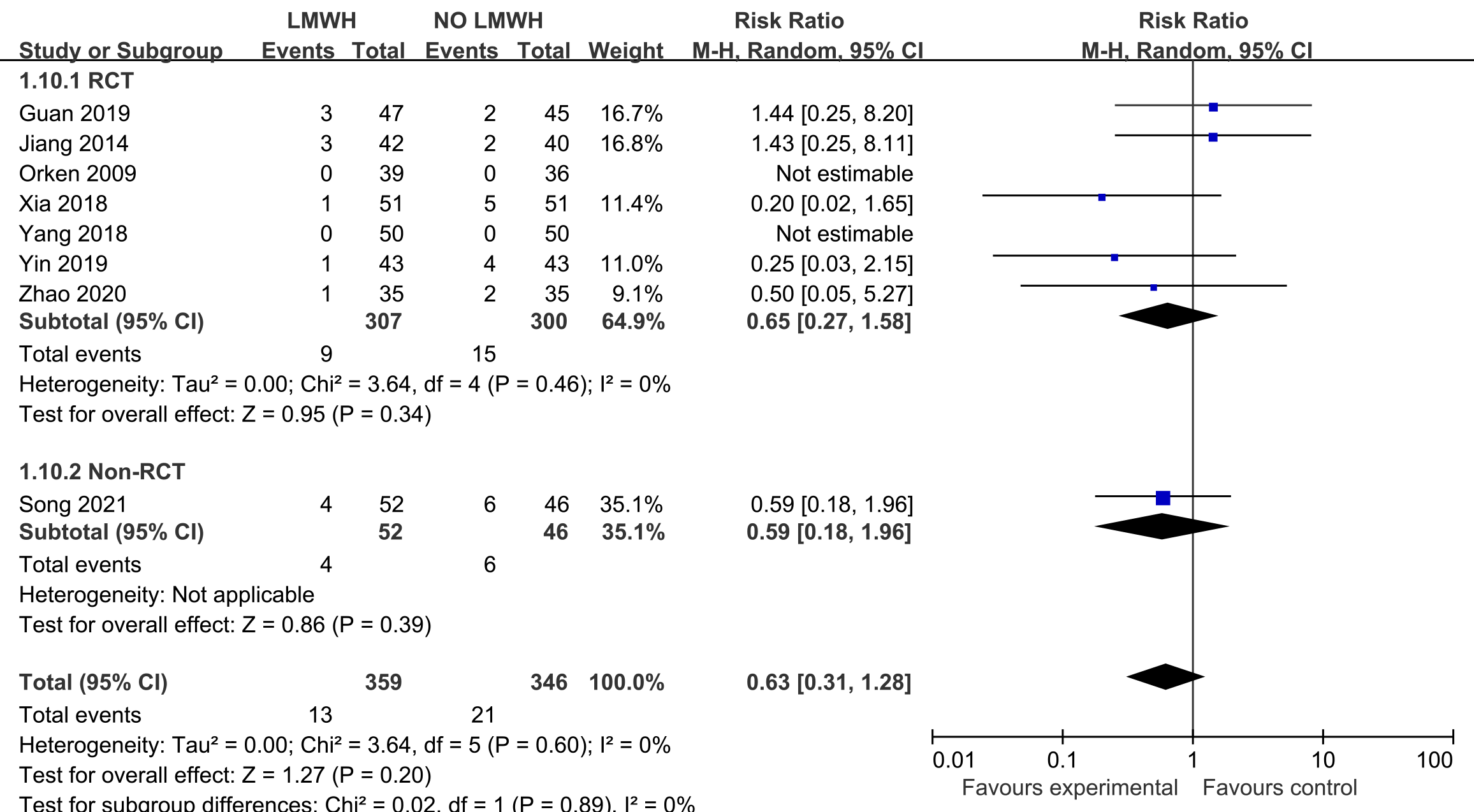

Supplement: S10 Fig — (TIF) [file pone.0311858.s016.tif]

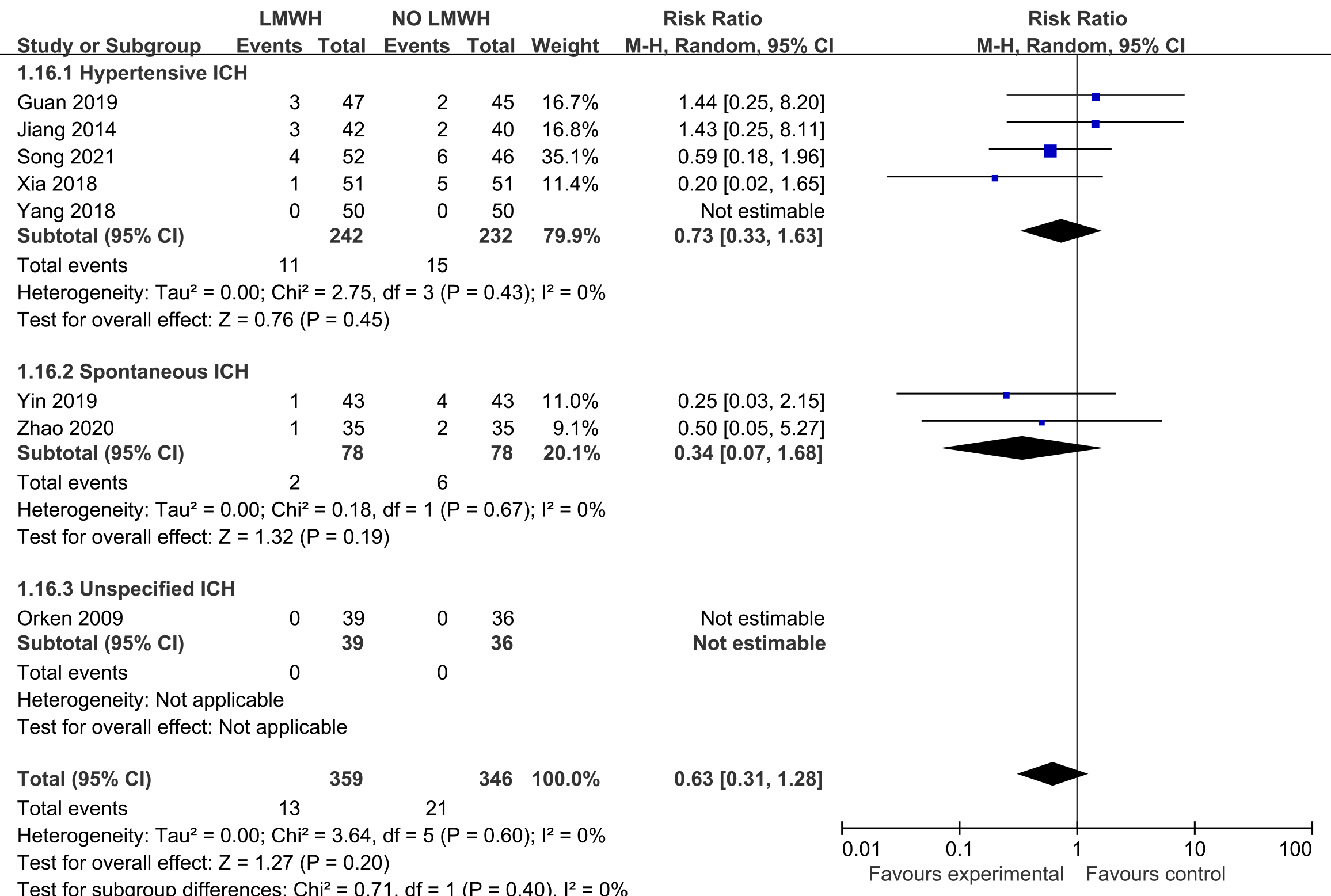

Supplement: S11 Fig — (TIF) [file pone.0311858.s017.tif]

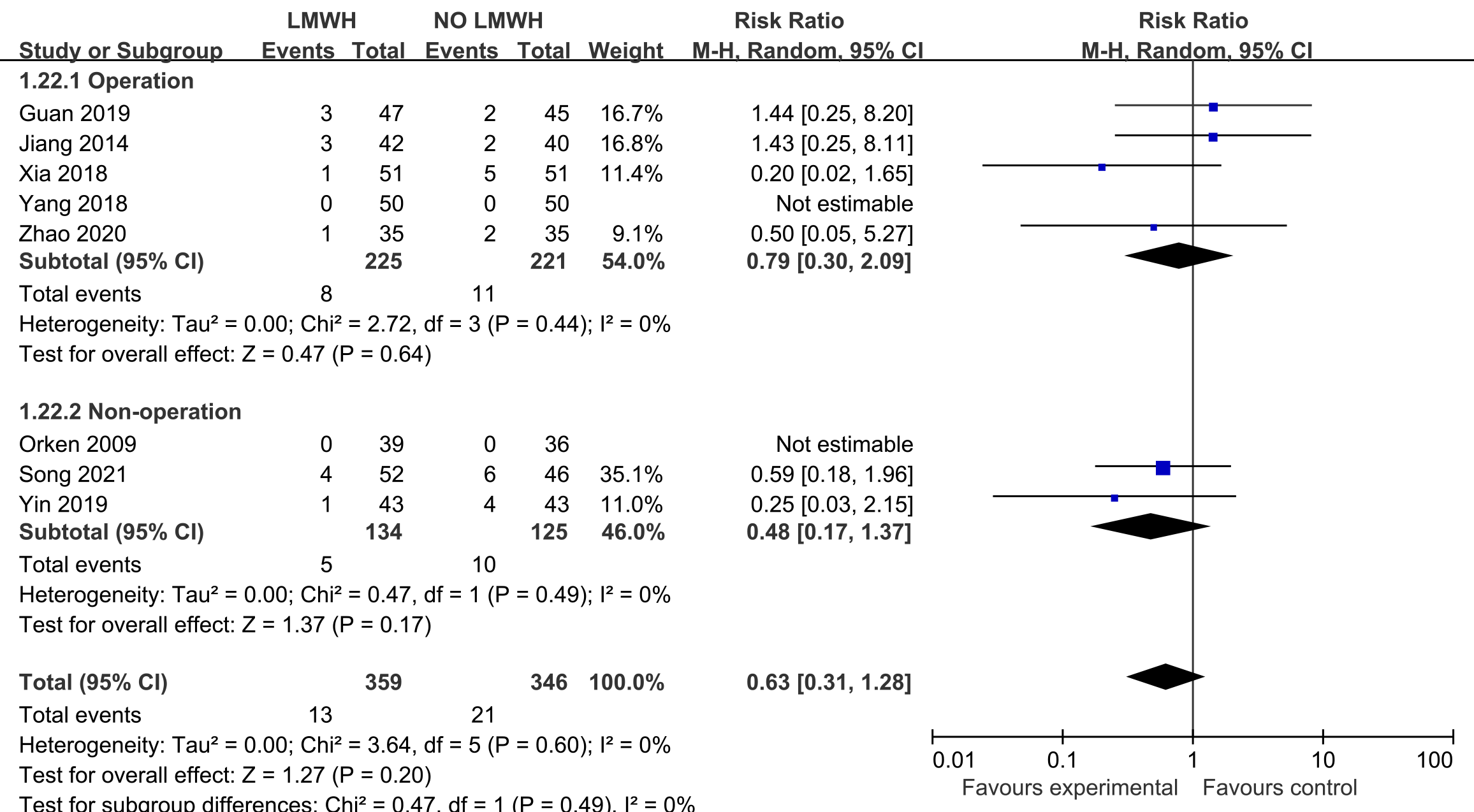

Supplement: S12 Fig — (TIF) [file pone.0311858.s018.tif]

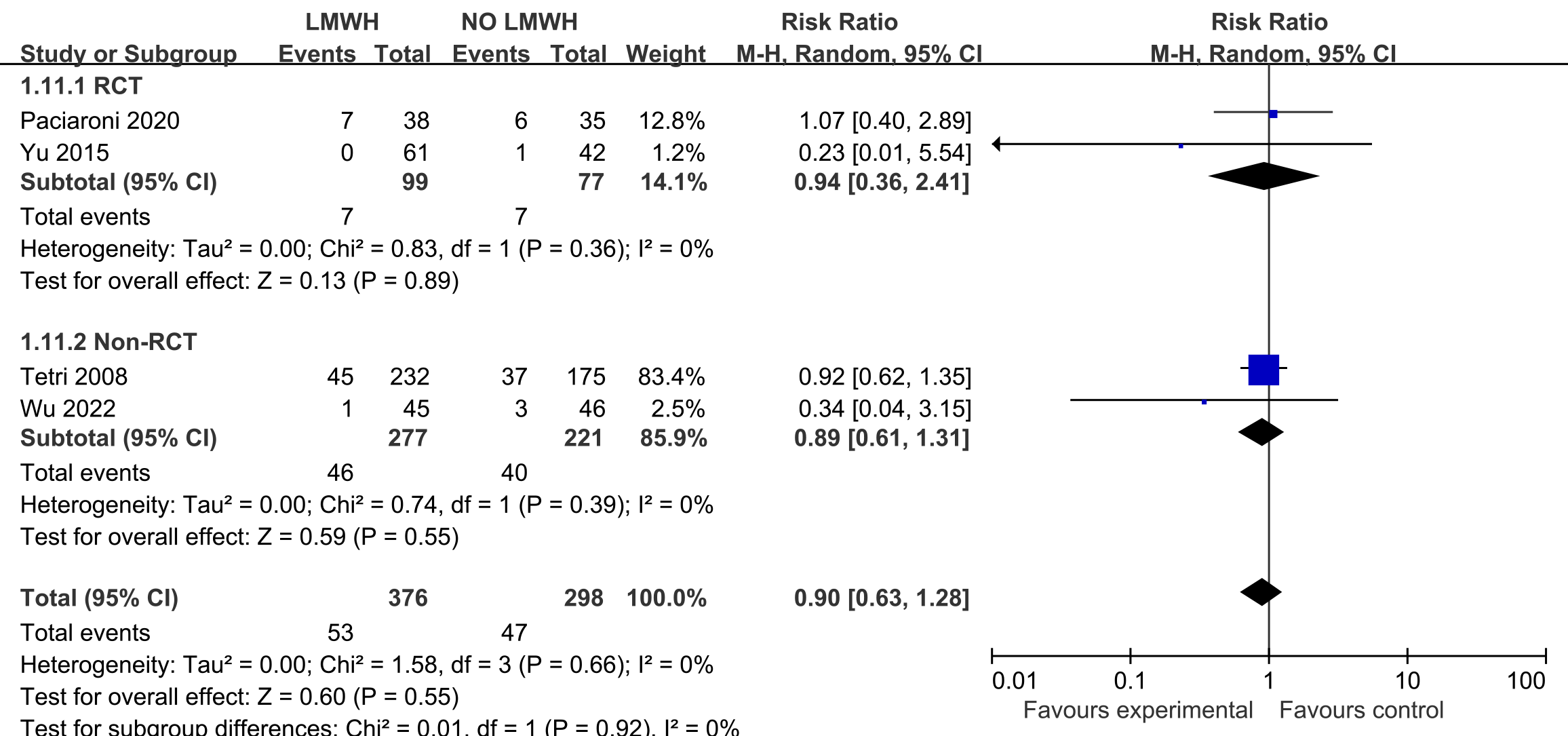

Supplement: S13 Fig — (TIF) [file pone.0311858.s019.tif]

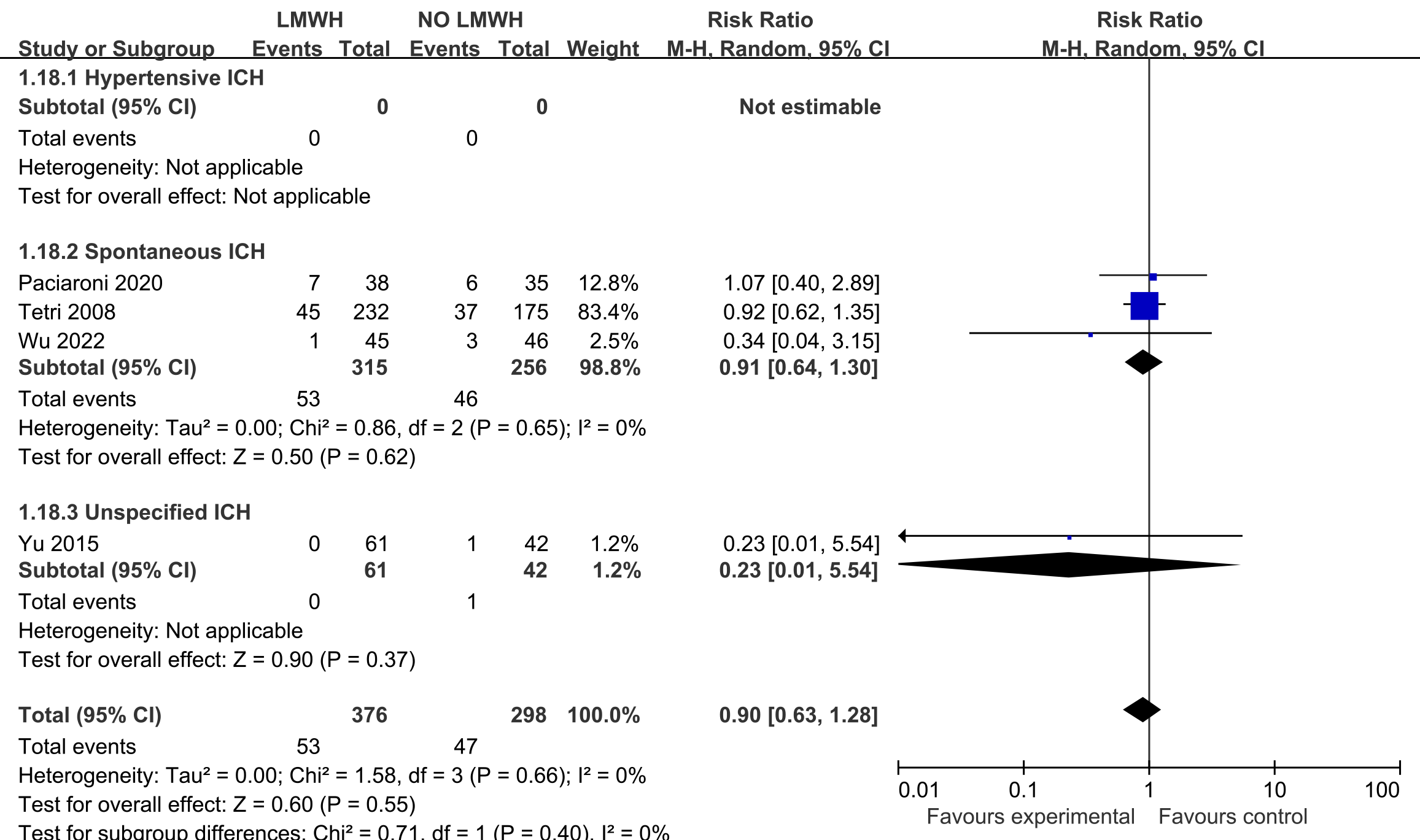

Supplement: S14 Fig — (TIF) [file pone.0311858.s020.tif]

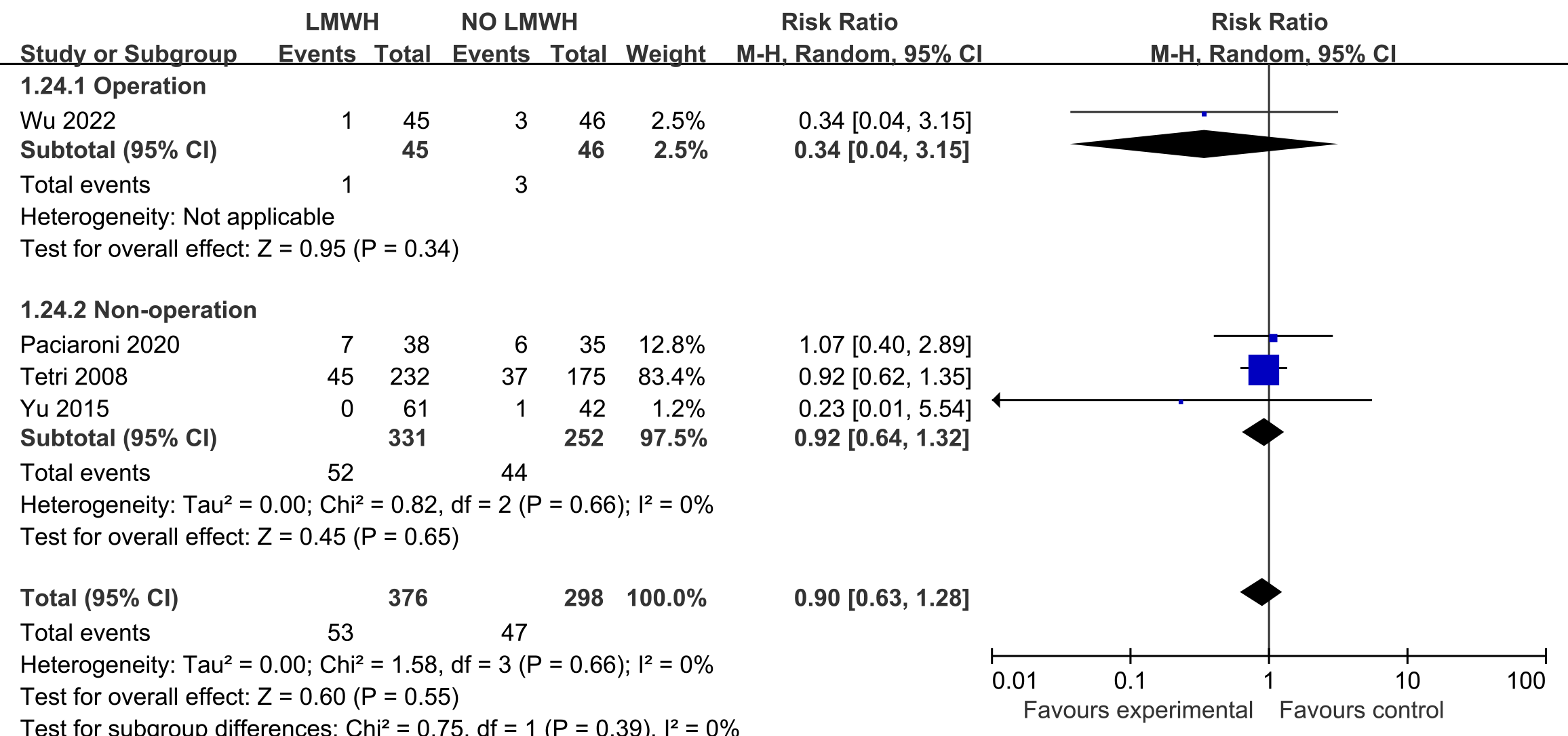

Supplement: S15 Fig — (TIF) [file pone.0311858.s021.tif]
