# Supplementary material for: Low-molecular-weight heparin in the prevention of venous thromboembolism among patients with acute intracerebral hemorrhage: A meta-analysis
Source: PLoS One. 2024 Oct 16;19(10):e0311858. doi: 10.1371/journal.pone.0311858 (PMC11482721; doi:10.1371/journal.pone.0311858)
Supplement: Attachment — Submitted filename: rebuttal letter.docx [file pone.0311858.s022.docx]

Dear Editors and Reviewers:

Thank you for your letter and for your comments concerning our manuscript entitled “Low-molecular-weight heparin in the prevention of venous thromboembolism among patients with acute intracerebral hemorrhage: A meta-analysis” (ID: PONE-D-24-20586). Those comments are all valuable and very helpful for revising and improving our paper, as well as the important guiding significance to our researches. We have studied comments carefully and have made correction which we hope meet with approval. The responds to your comments are as flowing:

**Responds to the academic editor’s comments:**

**Major comments:**

**1.** While the study attempts to address heterogeneity, the inclusion of both randomized controlled trials (RCTs) and non-randomized studies could introduce variability and affect the robustness of the conclusions. (Page 5, Lines 84-87).

**Response:** Thank you for your suggestion. It is really true as editor suggested that the inclusion of RCTs and non-RCTs could introduce variability and affect the robustness of the conclusions, so we conducted subgroup analysis of RCTS and non-RCTs respectively according to the type of study, and the results strengthened our findings.

**2.** The study focuses primarily on short-term outcomes, which may not fully capture the long-term safety and efficacy of LMWH in ICH patients (Page 9, Lines 188-190), thereby limiting the overall scope of this work.

**Response:** Thank you for your suggestion. As the editor noted, this is also a limitation of the included original studies, for which we regret the lack of long-term outcomes. If there are studies conducted to evaluate the long-time outcomes of LMWH on ICH patients in the future, we will conducted an improved meta-analysis.

**3.** The inclusion of non-randomized studies and concerns about overall bias in some studies need to be addressed more thoroughly (Page 5, Lines 84-87).

**Response:** Thank you for your suggestion. First, there was no obvious heterogeneity for the effect of LMWH on outcomes according to heterogeneity test. Second, we conducted subgroup analysis of RCTS and non-RCTs respectively according to the type of study to exclude the effect of inclusion of non-RCTs, and the results strengthened our findings. Third, we evaluated the quality of RCTs and non-RCTs with the revised Cochrane risk-of-bias tool (RoB 2) and Newcastle-Ottawa Scale respectively. Among the 21 RCTs, most articles showed some concerns in overall bias, only two were rated as high risk of overall bias. Among 9 non-RCTs, eight were adjudged to be high quality, only one was moderate quality. More large, multicenteric, and high-quality RCTs are needed for validating the findings and inform clinical practice.

**4.** While the protocol is registered in PROSPERO, additional details about deviations from the original protocol should be clearly stated (Page 4, Lines 58-59).

**Response:** Thank you for your suggestion. The details of our study are consistent with the original protocol registered in PROSPERO.

**5.** The exclusion criteria are appropriately detailed, but the justification for excluding specific types of studies (e.g., those involving other types of intracranial hemorrhage) could be better explained (Page 4, Lines 67-73).

**Response:** Thank you for your suggestion. We have re-written this part according to the editor’s suggestion. Studies involving other types of intracranial hemorrhage were excluded, because their pathogenesis are different from ICH.

**6.** The relatively short follow-up period in some included studies may limit the ability to observe long-term outcomes (Page 9, Lines 188-190)

**Response:** Thank you for your suggestion. As the editors noted, most of the studies we included were followed for 14 days, with only 4 studies followed for more than 3 months, which may affect the observation of long-term outcomes. If there are studies conducted to evaluate the long-time outcomes, we will conducted an improved meta-analysis.

**7.** The manuscript does not provide detailed practical recommendations for implementing LMWH treatment in clinical practice (Page 10, Lines 181-185)

**Response:** Thank you for your suggestion. According to the editor’s comments, we have made extensive modifitions to our manuscript.

**8.** The study does not compare the effectiveness of LMWH with other potential thromboprophylactic agents or methods, which could provide a more comprehensive clinical perspective (Page 3, Lines 52-53).

**Response:** Thank you for your nice comments on our article. Our study aimed to elucidate the role of LMWH for VTE prevention in ICH patients, so we compare the effectiveness and safety of prophylactic use of LMWH with no prophylactic use of LMWH. We will try it in the future.

**9.** There are a few typographical errors that need correction, such as "meta -analysis " instead of "meta-analysis" (Page 1, Line 1).

**Response:** Thank you for your suggestion. We have made correction according to the editor’s comments.

**10.** Ensure consistency in the use of abbreviations and terms throughout the manuscript, such as "RCT" vs. "non-RCT" (Page 5, Lines 84-85).

**Response:** Thank you for your suggestion. We have made correction according to the editor’s comments.

**11.** Some references are not up-to-date, and more recent studies could be included to support the findings (Page 10, Lines 198-307).

**Response:** Thank you for your suggestion. As suggested by the editor, we have added more up-to-date reference to support the findings.

**Minor comments:**

**12.** Abstract:
Line 1: Correct "meta -analysis " to "meta-analysis."

**Response:** Thank you for your suggestion. We have made correction according to the editor’s comments.
Line 15: Clarify the definition of "early low-dose" in the context of LMWH treatment.

**Response:** Thank you for your suggestion. We have made correction according to the editor’s comments in result and discussion sections.

**13.** Introduction:
Page 1, Line 2: Rephrase "life-threatening consequences, representing a significant global health burden" for clarity.

**Response:** Thank you for your suggestion. We have made correction according to the editor’s comments.
Page 2, Line 3: Provide more context on the current limitations of existing guidelines for LMWH use in ICH patients.

**Response:** Thank you for your suggestion. We have made correction according to the editor’s comments.

**14.** Methods:
Page 4, Line 55: Clarify the inclusion criteria for studies and justify the exclusion of non-original studies.

**Response:** Thank you for your suggestion. First, We formulated the inclusion criteria for studies in accordance with PICOS principles. Second, non-original studies can not be extracted with data.
Page 4, Line 67: Explain the rationale for excluding studies involving other types of intracranial hemorrhage.

**Response:** Thank you for your suggestion. Studies involving ICH patients caused by surgery, traumatic brain injury or patients with intracranial hemorrhage other than ICH were excluded, because their pathogenesis are different from ICH.

**15.** Results:

Page 7, Line 106: Elaborate on the reasons for excluding 22 articles after full-text assessment. These should also be included in the PRISMA diagram.

**Response:** Thank you for your suggestion. We have made correction according to the editor’s comments.

Page 8, Line 132: Provide more detail on the methods used to assess heterogeneity and the implications of the findings.

**Response:** Thank you for your suggestion. We have made correction according to the editor’s comments.

**16.** Discussion:
Page 9, Line 164: Discuss the clinical implications of the findings in more detail, including potential changes to clinical practice guidelines.

**Response:** Thank you for your suggestion. We have made correction according to the editor’s comments.
Page 10, Line 188: Address the limitations of the study more thoroughly, including the potential impact of short follow-up duration and inclusion of non-randomized studies.

**Response:** Thank for you suggestion. First, We conducted subgroup analysis of RCTS and non-RCTs respectively according to the type of study (supporting information), and the results strengthened our findings. Second, Most of the studies we included were followed for 14 days, with only 4 studies followed for more than 3 months. If there are studies conducted to evaluate the long-time effects of LMWH on ICH patients in the future, we will conducted an improved meta-analysis.

**17.** Conclusion:
Page 10, Line 190: Emphasize the need for large, multicentric, and high-quality RCTs to validate the findings and inform clinical practice.

**Response:** Thank you for your suggestion. We have made correction according to the editor’s comments.

**Responds to the reviewer’s comments:**

1. Abstract

-Please clarify the search strategy and inclusion criteria in the Methods section.

**Response:** Thank you for your suggestion. We have made correction according to the reviewer’s comments.

2. Methods section
   Search strategy and screening criteria

-Please state what designs of the studies were eligible for, including (RCT and cohort).

**Response:** Thank you for your suggestion. We have made correction according to the reviewer’s comments.

-Please clarify the type of ICH in the inclusion criteria to distinguish it from posttraumatic and postoperational.

**Response:** Thank you for your suggestion. We have made correction according to the reviewer’s comments.

-Please clarify if any automatic tools were used to screen titles and abstracts and exclude duplications.

**Response:** Thank you for your suggestion. We have made correction according to the reviewer’s comments.

3. Quality evaluation

-Due to the presence of different approaches for interpretation of the NOS score, please clarify your own.

**Response:** Thank you for your suggestion. We have made correction according to the reviewer’s comments.

4. Endpoints

-Please clarify the definitions of all outcomes. What is the difference between hematoma expansion and rebleeding?

**Response:** Thank you for your suggestion. We have made correction according to the reviewer’s comments. There is no difference between hematoma expansion and rebleeding.

-According to the analysis of the risk of selection bias, I can suggest that GI bleeding and hematoma expansion/rebleeding were not considered primary outcomes. Please clarify and divide outcomes into primary and secondary. Please note that all primary outcomes need a separate analysis for publication bias, as you already performed for DVT, PE, and hematoma expansion/rebleeding.

**Response:** Thank you for your suggestion. We have made correction according to the reviewer’s comments.

5. Statistical analysis

-Do you mean p<0.01 for heterogeneity?

**Response:** Yes.
-Please clarify a threshold for I2 that represents heterogeneity.

**Response:** Thank you for your suggestion. We have made correction according to the reviewer’s comments.
-Please clarify whether under treatment type you mean surgery or not surgery.

**Response:** Thank you for your suggestion. We have made correction according to the reviewer’s comments.
-Please consider subgroup analysis for the LMWH initiation time, which is crucial for

ICH.

**Response:** Thank you for your suggestion. LMWH was initiated on 3rd-4th days after hospitalization or ICH operation in most of studies, and only 2 studies started within 3 days, so it is difficult to perform subgroup analysis, but it is the direction of our next study.
-Also, it is interesting to look at the effect of LMWH in terms of different comparators (IPC alone, IPC + ESC, no prophylaxis).

**Response:** Thank you for your suggestion, We will try it in the future.

6. Results
   Study screening

-What do you mean by the use of heparin in the excluded studies? Unfractionated heparin? Please consider that you do not have such an exclusion criterion in the methods section

**Response:** Thank you for your suggestion, studies using of unfractionated heparin were excluded. We have made correction in methods section.

7. Study outcomes

-You stated about the non-significant increase in GI bleeding; however, the forest plot favors LMWH. Please check this statement.

**Response:** Thank for your suggestion, we have check this statement. Our statement is consistent with the forest plot. The LMWH group showed a non-significant increase in GI bleeding (3.6% vs. 6.1%; RR, 0.63; 95% CI, 0.31–1.28; p-value= 0.20).

8. Discussion

-Please discuss the clinical relevance of your findings in the context of the lack of influence of LMWH on mortality.

**Response:** Thank for your suggestion. We have made correction according to the reviewer’s comments.

9. Figure 1

Please design a flowchart in accordance with the PRISMA standard and describe what other sources were used to find 3 records (reference list of relevant papers? authors' archive?)

**Response:** Thank for your suggestion. We have made correction according to the reviewer’s comments.

10. Supplementary

-Please check your query. Using #1 for Pubmed, I found 60280 results that significantly exceed the stated 13451.

**Response:** Thank for your suggestion, We have checked our query. Instead of just using #1, you can try to use #1-#7.

We tried our best to improve the manuscript and made some changes in the manuscript. These changes will not influence the content and framework of the paper. And here we did not list the changes but marked in red in revised paper. We appreciate for Editors/Reviewers’ warm work earnestly, and hope that the correction will meet with approval. Once again, thank you very much for your comments and suggestions.

Best regards!

Yours sincerely,

Corresponding author: Haizheng Li

E-mail: lihaizhengbdyy@163.com
