# Supplementary material for: Low-molecular-weight heparin in the prevention of venous thromboembolism among patients with acute intracerebral hemorrhage: A meta-analysis
Source: PLoS One. 2024 Oct 16;19(10):e0311858. doi: 10.1371/journal.pone.0311858 (PMC11482721; doi:10.1371/journal.pone.0311858)
Supplement: Attachment — Submitted filename: rebuttal letter (Response to Reviewers).docx [file pone.0311858.s023.docx]

Dear Editors and Reviewers:

Thank you for your letter and for your comments concerning our manuscript entitled “Low-molecular-weight heparin in the prevention of venous thromboembolism among patients with acute intracerebral hemorrhage: A meta-analysis” (ID: PONE-D-24-20586R1). Those comments are all valuable and very helpful for revising and improving our paper, as well as the important guiding significance to our researches. We have studied comments carefully and have made correction which we hope meet with approval. The responds to your comments are as flowing:

**Responds to the reviewer #3’s comments:**

1. The meta-analysis inclusion criteria only states: studies reporting patients with ICH. It should be corrected to spontaneous ICH as other ICH types are listed in the exclusion criteria.

**Response:** Thank you for your suggestion. Our meta-analysis enrolled studies included ICH patients (hypertensive ICH, spontaneous ICH, and unspecified ICH), not only spontaneous ICH, and we performed subgroup analyses based on ICH type. “Other ICH” in exclusion criteria included ICH caused by surgery, traumatic brain injury, or those with intracranial hemorrhages other than ICH (e.g., subarachnoid hemorrhage, traumatic intracerebral hemorrhage, subdural hematoma, or epidural hematoma).

2. Hematoma expansion and rebleeding are basically the same. I would recommend using the term “hematoma progression” instead. Also correct in Table 1

**Response:** Thank you for your suggestion. We have made correction according to the reviewer’s comments.

3. In the Results section: four studies were excluded due to heparin use. I’m guessing you mean unfractionated heparin. Also correct in Figure 1.

**Response:** Thank you for your suggestion. We have made correction according to the reviewer’s comments.

4. Please elaborate why did you include 11 studies with unspecified ICH if your primary focus was on spontaneous ICH

**Response:** Thank you for your suggestion. Our primary focus was on ICH, not only on spontaneous ICH, and we performed subgroup analyses based on ICH type.

5. The risk of GI bleeding was non-significantly lower in the LMWH group based on the reported numbers and Forest plot. Please correct in the Results section.

**Response:** Thank you for your suggestion. We thought “a non-significant rise” more adequate, because GI is one of complications.

**Responds to the reviewer #4’s comments:**

**Response:** Thank you for your suggestion. Our search strategy did not miss ICH, it is in supporting information (S1 Table. Search Details #1). Hematoma progression is primary outcome, but GI is secondary outcome, We will evaluate a composite outcome of any major bleeding in the future.

We tried our best to improve the manuscript and made some changes in the manuscript. These changes will not influence the content and framework of the paper. And here we did not list the changes but marked in red in revised paper. We appreciate for Editors/Reviewers’ warm work earnestly, and hope that the correction will meet with approval. Once again, thank you very much for your comments and suggestions.

Best regards!

Yours sincerely,

Corresponding author: Haizheng Li

E-mail: lihaizhengbdyy@163.com
